# Multi-layer transcriptomic analyses identify a mucin-associated epithelial program linked to innate inflammatory injury in ulcerative colitis

**DOI:** 10.3389/fimmu.2026.1846672

**Published:** 2026-06-03

**Authors:** Jianmin Huang, Jingui He, Haimin Li, Rongrong Chen, Peimin Li, Jiangchong Zhao, Xin Gao

**Affiliations:** 1 Department of Digestive Endoscopy, Fuzhou University Affiliated Provincial Hospital, Fuzhou, Fujian, China; 2 Shenzhen Hospital (Futian) of Guangzhou University of Chinese Medicine, Shenzhen, China; 3 The Sixth Clinical Medical College of Guangzhou University of Traditional Chinese Medicine, Shenzhen, China; 4 Department of Gastroenterology, Fuzhou University Affiliated Provincial Hospital, Fuzhou, Fujian, China; 5 Shengli Clinical Medical College of Fujian Medical University, Fuzhou, Fujian, China; 6 Department of Gastroenterology, The Fifth Clinical Medical College of Shanxi Medical University, Taiyuan, Shanxi, China; 7 Department of Gastroenterology, The Second Hospital of Hebei Medical University, Shijiazhuang, Hebei, China

**Keywords:** epithelial-immune crosstalk, IL-18, IL-1β, innate inflammatory injury, mucin-type O-glycosylation, single-cell RNA sequencing, spatial transcriptomics, ulcerative colitis

## Abstract

Epithelial barrier disruption and mucosal innate immune activation reinforce each other in ulcerative colitis (UC), yet the epithelial glycosylation programs linking mucus homeostasis to inflammatory tissue injury remain incompletely defined. Here, we integrated bulk, single-cell, and spatial transcriptomic analyses with functional validation to identify mucin-associated epithelial determinants relevant to UC. In the discovery cohort GSE107499, 15 dysregulated mucin-type O-glycosylation and mucin-associated genes were identified and were enriched mainly in O-glycan biosynthesis, glycosyltransferase activity, and Golgi-associated secretory functions. An integrative prioritization framework highlighted GALNT12 as the leading candidate. Low GALNT12 expression was associated with enrichment of TNFA signaling via NF-kB, inflammatory response, interferon gamma response, IL6-JAK-STAT3 signaling, and complement, together with higher infiltration scores for monocyte lineage cells, neutrophils, fibroblasts, and T-cell populations. At single-cell resolution, GALNT12 was concentrated in epithelial cells, particularly MUC2-positive epithelial cells. MUC2-positive epithelial cells with detectable GALNT12 expression showed broader inferred incoming and outgoing communication with surrounding epithelial, stromal, and immune populations and were enriched for proteostasis and secretory trafficking programs. Spatial transcriptomic analysis further identified GALNT12-high mucin-associated niches that co-localized with stronger goblet and mucin signatures and lower inflammatory scores. In HT29-19A cells, GALNT12 knockdown exacerbated TNF-α-induced LDH release, reduced OCLN and TJP1 expression, increased IL-8, IL-6, CCL2, IL-1β, and IL-18 production, and enhanced caspase-1 processing detected as cleaved caspase-1 p10 together with increased GSDMD-N formation under TNF-α stress. Collectively, these findings identify a GALNT12-centered mucin-associated epithelial program linked to epithelial barrier resilience and relatively restrained innate inflammatory injury at the barrier–immune interface in UC, while supporting a caspase-1/GSDMD-associated inflammatory injury phenotype under cytokine stress.

## 1 Introduction

Ulcerative colitis (UC) is a chronic inflammatory disorder of the colonic mucosa sustained by complex interactions among epithelial injury, dysregulated immunity, and microbial exposure (1, 2). In addition to adaptive immune remodeling, persistent disease activity reflects a failure of the epithelial barrier to contain luminal triggers that can amplify innate inflammatory responses (3). A major component of this defense system is the colonic mucus layer, which physically separates luminal bacteria from the epithelial surface (4). This barrier is primarily built by the highly O-glycosylated mucin MUC2. In active UC, the mucus barrier undergoes early structural weakening and allows bacterial penetration (5, 6). Evidence indicates that tissue inflammation is intimately connected to modifications in MUC2 O-glycans (7). Such findings imply that glycan remodeling and disruption of mucin biosynthesis may not simply be passive secondary effects of ulcerative colitis. Instead, these changes may contribute to epithelial susceptibility and facilitate the expansion of inflammatory responses.

Epithelial glycosylation plays an important role in ulcerative colitis and is critical for barrier-immune integrity and secretory homeostasis. The UDP-GalNAc:polypeptide N-acetylgalactosaminyltransferase family (8) initiates mucin-type O-glycosylation, and by virtue of its preferential expression in digestive tissues and specialized activity toward mucin-type substrates (9, 10), GALNT12 has potential digestive tissue function. Studies have shown that GALNT12 is functionally implicated in colorectal epithelial biology and cancer susceptibility (11, 12). However, its relationship with UC-associated epithelial inflammatory injury, mucin-associated epithelial remodeling, and epithelial–immune communication remains insufficiently defined. Since glycosylation status contributes to the glycoprotein composition and functional organization of the mucosal surface, clarifying this relationship may help explain how epithelial glycosylation programs connect mucus barrier integrity with innate inflammatory responses in UC.

Modern single-cell transcriptomic investigations have provided a detailed atlas of the cellular environment in ulcerative colitis, revealing not only significant diversity within the epithelium but also extensive remodeling across the stromal, myeloid, and adaptive immune compartments (13–17). Even so, mucin-type O-glycosylation has not been systematically examined through an integrated framework that connects epithelial states, tissue spatial organization, and inflammatory injury phenotypes. In the present study, we combined multi-layer transcriptomic analyses with functional validation to identify a mucin-associated epithelial program linked to inflammatory remodeling in UC. We found that GALNT12 was consistently prioritized across analytical layers. Importantly, our data indicate that reduced GALNT12 expression is associated with altered epithelial-immune communication, a stronger innate-inflammatory tissue context, and increased susceptibility to cytokine-driven epithelial injury.

## 2 Materials and methods

### 2.1 Study design and public datasets

Publicly available transcriptomic datasets were obtained from the Gene Expression Omnibus. Bulk transcriptomic analyses used GSE107499 as the discovery cohort. According to the GEO record, GSE107499 comprises inflamed and non-inflamed colonic biopsies from subjects with ulcerative colitis; therefore, the discovery comparison was based on inflamed versus non-inflamed ulcerative colitis tissue rather than healthy controls versus ulcerative colitis. GSE47908 and GSE87466 were used as external validation cohorts. Single-cell RNA sequencing analysis used GSE214695. Because GSE214695 is a broader inflammatory bowel disease dataset, only healthy control and ulcerative colitis samples were retained for the analyses reported here. Spatial transcriptomic analysis used GSE189184, and seven samples (B4, B5, B8, B9, B12, B13, and C2) were analyzed. A predefined mucin-type O-glycosylation and mucin-associated gene set was curated before analysis. The candidate set included mucins and genes related to mucin-type O-glycosylation, including glycosylation enzymes involved in the initiation and remodeling of mucin-type O-glycans. The genes included in this candidate set are listed in **Supplementary Table 1**. *In vitro* validation was performed in the human colorectal epithelial subclone HT29-19A (HT29/C1.19A).

### 2.2 Identification of differentially expressed mucin-type O-glycosylation and mucin-associated genes

Differential expression analysis in GSE107499 was performed between inflamed and non-inflamed colonic biopsies from patients with ulcerative colitis using the limma package (18). Genes with an absolute log2 fold change greater than 0.585 and an adjusted P value less than 0.05 were defined as differentially expressed genes. The overlap between the differentially expressed genes and the predefined mucin-type O-glycosylation-related gene set was retained for downstream analyses. Normalized expression profiles of the overlapping genes were visualized by heatmap with unsupervised hierarchical clustering.

### 2.3 Protein-protein interaction analysis, enrichment analysis, and functional prioritization

Protein-protein interaction analysis of the intersecting genes was performed using STRING with a minimum interaction score of 0.4 (19). Gene Ontology biological process, cellular component, and molecular function analyses, together with Kyoto Encyclopedia of Genes and Genomes enrichment analyses, were conducted using clusterProfiler (20). Terms with adjusted P values less than 0.05 were considered enriched. Functional prioritization was further performed by Gene Ontology semantic similarity analysis using GOSemSim (21). Genes with higher semantic similarity scores were considered more functionally central within the candidate gene set.

### 2.4 Machine learning model comparison and SHAP interpretation

The 15 candidate genes were subjected to systematic model comparison across multiple feature-selection and classification algorithms. Expression matrices were harmonized across cohorts, with duplicated gene symbols averaged. GSE107499 served as the discovery/training set, and GSE47908 and GSE87466 as external validation sets. Features were standardized using training-cohort parameters and applied to validation sets. Models—including penalized regression (ridge, lasso, elastic net), tree-based methods (random forest, boosting, XGBoost), support vector machine, naive Bayes, partial least squares, and linear discriminant analysis—were constructed in R using caret, glmnet, xgboost, and related packages. Performance was assessed by the area under the receiver operating characteristic curve (AUC), with internal five-fold cross-validation and external validation in independent cohorts. The optimal classifier was selected, and SHAP analysis (22) was performed to quantify each gene’s contribution to the predicted probability of disease or inflammation, visualized by global importance, dependence, and waterfall plots.

### 2.5 GALNT12-defined subgroup analysis in bulk transcriptomic data

GALNT12 was selected for downstream analyses based on semantic similarity ranking, model interpretation, and biological relevance to mucin-type O-glycosylation. Ulcerative colitis samples were stratified into GALNT12-high and GALNT12-low groups according to the median GALNT12 expression level. Differential expression analysis between subgroups was performed using limma (18). Hallmark gene set enrichment analysis was conducted using the Molecular Signatures Database Hallmark collection, and results were interpreted according to the normalized enrichment score and adjusted P value (23). Immune and stromal cell infiltration was estimated using MCPcounter (24).

### 2.6 Single-cell RNA sequencing processing and cell annotation

ScRNA-seq data from GSE214695 were analyzed using Seurat (25). Raw 10x matrices were imported with min.cells = 3 and min.features = 100, and cells meeting quality-control criteria (nFeature_RNA > 50, percent.mt < 15%) were retained. Data were normalized (LogNormalize, scale factor 10,000), variable features were selected by the vst method (nfeatures = 2,000), and the expression matrix was scaled for PCA (30 PCs). Batch effects were corrected using Harmony (orig.ident, dimensions 1–30). Clustering (resolution 0.5), UMAP, and t-SNE were performed on the Harmony-aligned dimensions, yielding major epithelial, stromal, and immune populations that were manually annotated using canonical markers. GALNT12 expression was summarized across annotated cell types, and the MUC2-positive epithelial subset was extracted for downstream analysis.

### 2.7 Cell-cell communication analysis in GALNT12-defined epithelial subsets

For the analysis of intercellular signaling within the ulcerative colitis epithelial MUC2-positive population, individual cells were categorized based on their GALNT12 expression status. Cells displaying detectable counts of GALNT12 were designated as GALNT12-positive (referred to as 12pos), while those lacking measurable GALNT12 transcripts were grouped as GALNT12-negative (referred to as 12neg). Cell-cell communication analysis was performed using CellChat with the human ligand-receptor database. Secreted signaling interactions were retained, communication probabilities were computed, and interactions were filtered using min.cells = 10 (26). Incoming and outgoing communication strengths were summarized by aggregating inferred communication probabilities across interacting partners. Bubble plots and circle plots were generated to visualize the breadth and strength of cell-cell communication.

### 2.8 Differential expression and virtual knockout analyses in MUC2-positive epithelial cells

For endogenous GALNT12-associated transcriptional analysis, only ulcerative colitis epithelial MUC2-positive cells were retained. Differential expression between 12pos and 12neg cells was computed with Seurat FindMarkers using the Wilcoxon rank-sum test, with min.pct = 0.05, logfc.threshold = 0, and return.thresh = 1. Significant genes were defined by adjusted P values less than 0.05 and absolute average log2 fold change greater than 0.25. For perturbational analysis, virtual knockout of GALNT12 was performed in healthy control-derived, GALNT12-positive, MUC2-positive epithelial cells using scTenifoldKnk to model perturbation from a less inflamed baseline and minimize confounding by UC-associated inflammatory remodeling. Selected cells were normalized, the top 3,000 highly variable genes were identified, and these genes—together with GALNT12 and mitochondrial genes—were used to build the regulatory input matrix. Ten sub-networks were generated by random sampling of up to 500 cells per network, and differentially perturbed genes were extracted from the diffRegulation output. Genes with adjusted P values < 0.05 were selected for downstream enrichment analysis. This virtual knockout analysis was interpreted as hypothesis-generating rather than direct biochemical evidence (27).

### 2.9 Spatial transcriptomic analysis and construction of a GALNT12-centered niche

Spatial transcriptomic data from GSE189184 were analyzed across seven samples, including B4, B5, B8, B9, B12, B13, and C2. For each sample, data were processed independently using the Spatial assay. To enrich for mucin-associated epithelial regions, a predefined focus signature composed of MUC2, EPCAM, KRT8, KRT18, TFF3, and FCGBP was scored with AddModuleScore. Spots above the 50th percentile of this score were defined as focus spots. Within focus spots, GALNT12 expression was spatially smoothed using a nearest-neighbor strategy with k = 6 and self-weight = 6. Among focus spots with positive smoothed GALNT12 signal, the median value was used to define GALNT12-high and GALNT12-low niches. Goblet and mucin program, epithelial injury, and inflammation core signatures were then evaluated by AddModuleScore. Sample-level median scores were compared with paired Wilcoxon tests.

### 2.10 Cell culture, siRNA transfection, and inflammatory stimulation

HT29-19A (HT29/C1.19A) cells were cultured in McCoy’s 5A medium containing 10% fetal bovine serum, 100 U/mL penicillin, and 100 μg/mL streptomycin at 37 °C in 5% CO2. The cell line was obtained from the Cell Bank of the Chinese Academy of Sciences (Shanghai, China), and was routinely tested and confirmed to be free of mycoplasma contamination. Cells were transfected with GALNT12-targeting siRNA or negative control siRNA using Lipofectamine 3000. At 24 h after transfection, cells were treated with or without 10 ng/mL recombinant human TNF-α to establish an inflammatory barrier injury model. Four groups were studied: si-NC, siGALNT12, si-NC + TNF-α, and siGALNT12 + TNF-α.

### 2.11 Western blotting, RT-qPCR, LDH release assay, and ELISA

Total protein was extracted with RIPA lysis buffer and analyzed by Western blotting using antibodies against GALNT12, caspase-1, GSDMD-N, and β-actin. Total RNA was extracted with TRIzol, reverse transcribed into cDNA, and analyzed by SYBR Green-based quantitative PCR. ACTB was used as the endogenous control, and relative mRNA expression was calculated using the 2^(-ΔΔCt)^ method. Inflammatory membrane injury was assessed by measuring lactate dehydrogenase (LDH) release in culture supernatants according to the manufacturer’s instructions. Culture supernatants were also collected for enzyme-linked immunosorbent assay measurement of IL-8, IL-6, CCL2, IL-1β, and IL-18. Detailed information regarding the key reagents, antibodies, commercial kits, and synthetic primer sequences used in this study is summarized in **Supplementary Table 2** and **Supplementary Table 3**.

### 2.12 Statistical analysis

Unless otherwise stated, data are presented as mean ± SD. For comparisons between two groups, two-tailed unpaired Student’s t-tests were used. For comparisons involving more than two groups, analysis of variance with appropriate *post hoc* multiple-comparison correction was used. Paired spatial comparisons were analyzed with paired Wilcoxon tests. Transcriptomic group comparisons were performed using the statistical procedures implemented in the corresponding R packages, with multiple-testing correction where appropriate. A two-sided P value less than 0.05 was considered statistically significant.

## 3 Results

### 3.1 Fifteen dysregulated mucin-type O-glycosylation and mucin-associated genes were identified in ulcerative colitis

**Figure 1:**
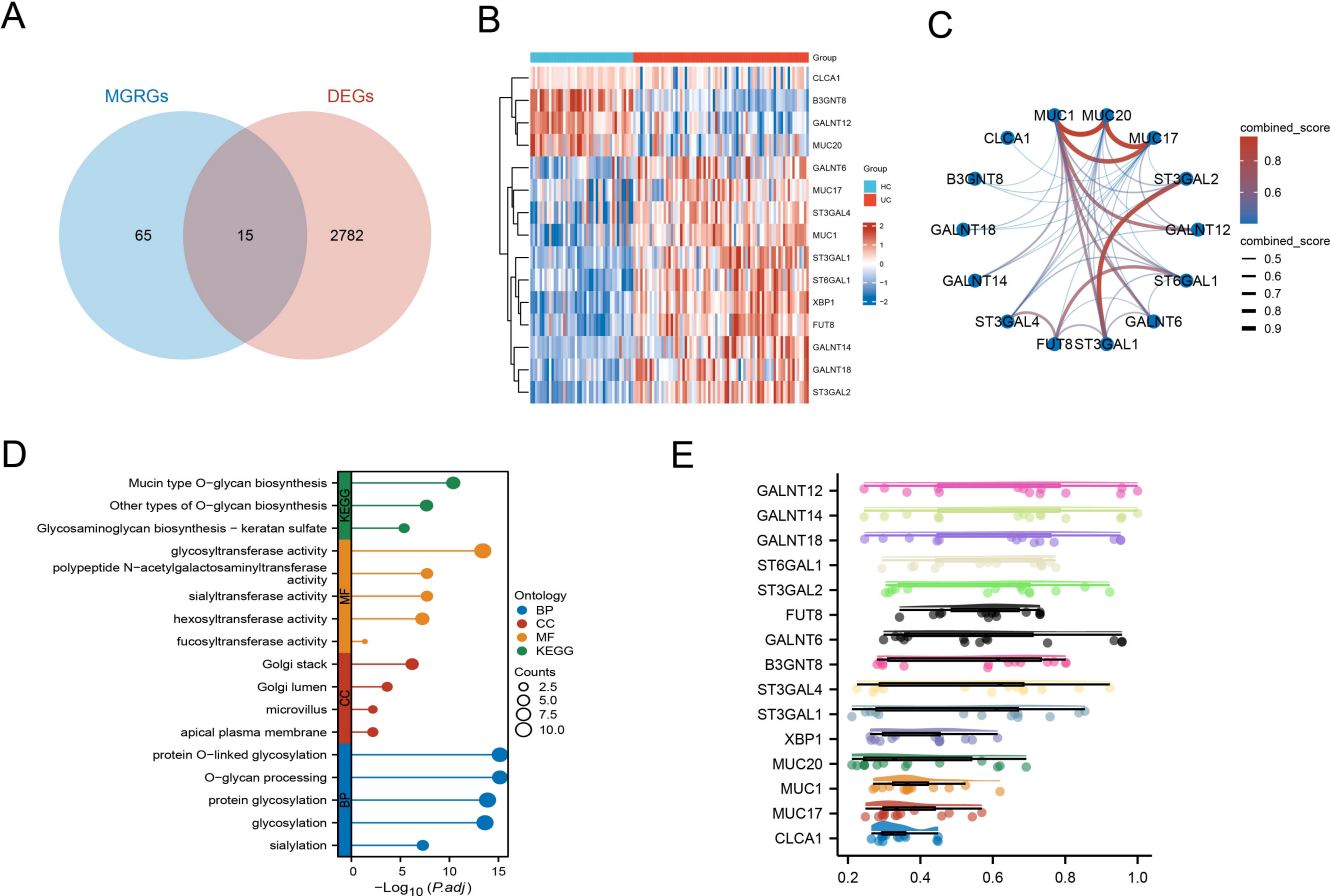
Identification and functional characterization of differentially expressed mucin-type O-glycosylation-related genes in the GSE107499 discovery cohort. **(A)** Venn diagram showing the overlap between mucin-type O-glycosylation-related genes and differentially expressed genes, yielding 15 intersecting genes. **(B)** Heatmap showing the expression profiles of the 15 intersecting genes in inflamed and non-inflamed colonic biopsies from the GSE107499 ulcerative colitis discovery cohort. **(C)** Protein-protein interaction network of the 15 intersecting genes constructed using STRING. Edge color and thickness indicate interaction confidence. **(D)** Gene Ontology and KEGG enrichment analyses of the 15 intersecting genes. Dot size represents gene count. **(E)** Semantic similarity-based functional prioritization of the 15 intersecting genes.

In the GSE107499 discovery cohort, differential expression analysis comparing inflamed and non-inflamed colonic biopsies from ulcerative colitis subjects identified 2,797 differentially expressed genes. Intersecting these genes with the predefined mucin-type O-glycosylation-related gene set yielded 15 candidate genes, including CLCA1, B3GNT8, GALNT12, MUC20, GALNT6, MUC17, ST3GAL4, MUC1, ST3GAL1, ST6GAL1, XBP1, FUT8, GALNT14, GALNT18, and ST3GAL2 (**Figure 1A**).

The heatmap showed a clear expression contrast between inflamed and non-inflamed ulcerative colitis biopsies, and unsupervised clustering generally separated the two groups (**Figure 1B**). CLCA1, B3GNT8, GALNT12, and MUC20 were among the lower-expression genes, whereas GALNT6, MUC17, ST3GAL4, MUC1, ST3GAL1, ST6GAL1, XBP1, FUT8, GALNT14, GALNT18, and ST3GAL2 were relatively increased. The protein-protein interaction network revealed extensive connectivity among mucins and glycosylation enzymes, suggesting coordinated remodeling of the mucin glycosylation machinery rather than isolated gene-level perturbations (**Figure 1C**).

Functional enrichment analysis showed that these genes were mainly involved in glycosylation, protein glycosylation, O-glycan processing, protein O-linked glycosylation, and sialylation in the biological process category, together with Golgi stack, Golgi lumen, microvillus, and apical plasma membrane in the cellular component category (**Figure 1D**). The molecular function category was dominated by glycosyltransferase activity, polypeptide N-acetylgalactosaminyltransferase activity, sialyltransferase activity, hexosyltransferase activity, and fucosyltransferase activity. KEGG analysis highlighted mucin-type O-glycan biosynthesis, other types of O-glycan biosynthesis, and glycosaminoglycan biosynthesis. Semantic similarity analysis further prioritized GALNT12, GALNT14, GALNT18, ST6GAL1, and ST3GAL2 among the 15 candidates (**Figure 1E**).

### 3.2 Integrative prioritization highlighted GALNT12 as a leading epithelial glycosylation determinant

**Figure 2:**
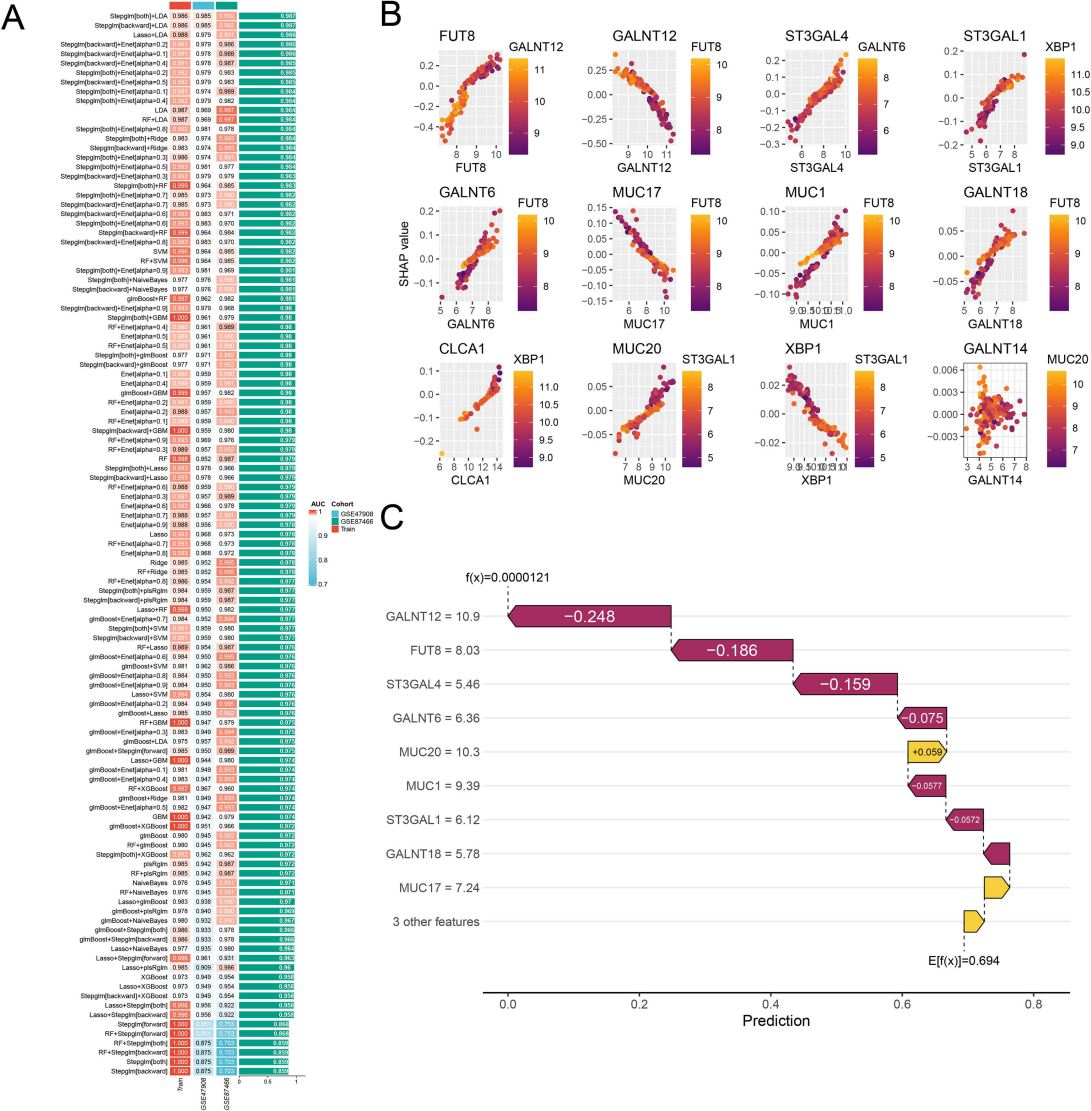
Machine-learning model comparison and SHAP-based interpretation of candidate mucin-type O-glycosylation-related genes. **(A)** Heatmap showing area under the curve values of different machine-learning model combinations in the discovery cohort and external validation cohorts. The right-side bar indicates the mean area under the curve across cohorts. **(B)** SHAP dependence plots showing the effects of representative genes on model output. Each dot represents one sample, and color indicates the interacting feature value. **(C)** SHAP waterfall plot illustrating feature-level contributions to the prediction of a representative sample.

To evaluate whether the candidate glycosylation genes carried robust disease-associated information, we compared multiple machine-learning algorithm combinations across the discovery cohort and two independent validation cohorts. Several models performed well, but the combination of bidirectional stepwise generalized linear modeling and linear discriminant analysis showed the most consistent cross-cohort performance, with area under the curve values of 0.986 in the discovery cohort, 0.985 in GSE47908, and 0.992 in GSE87466, corresponding to a mean area under the curve of 0.987 (**Figure 2A**). These findings indicate that the dysregulated mucin-type O-glycosylation gene set captures a reproducible transcriptomic signal associated with UC tissue status.

Based on the SHapley Additive exPlanations dependence plots, several genes—including FUT8, GALNT12, ST3GAL4, GALNT6, ST3GAL1, XBP1, MUC17, MUC1, GALNT18, CLCA1, MUC20, and GALNT14—were identified as major contributors to the model output, frequently exhibiting non-linear effects (**Figure 2B**). Analysis of a representative waterfall plot further demonstrated that GALNT12, FUT8, ST3GAL4, and GALNT6 exerted the most substantial influence on the final prediction score, while MUC20 provided a minor contribution in the opposing direction (**Figure 2C**). We selected GALNT12 for subsequent mechanistic investigations because it consistently ranked high in semantic similarity analysis, played a central role in model interpretation, and possesses a well-established biological connection to mucin-type O-glycosylation within digestive tissues.

### 3.3 Low GALNT12 expression marked an innate-inflammatory tissue context

**Figure 3:**
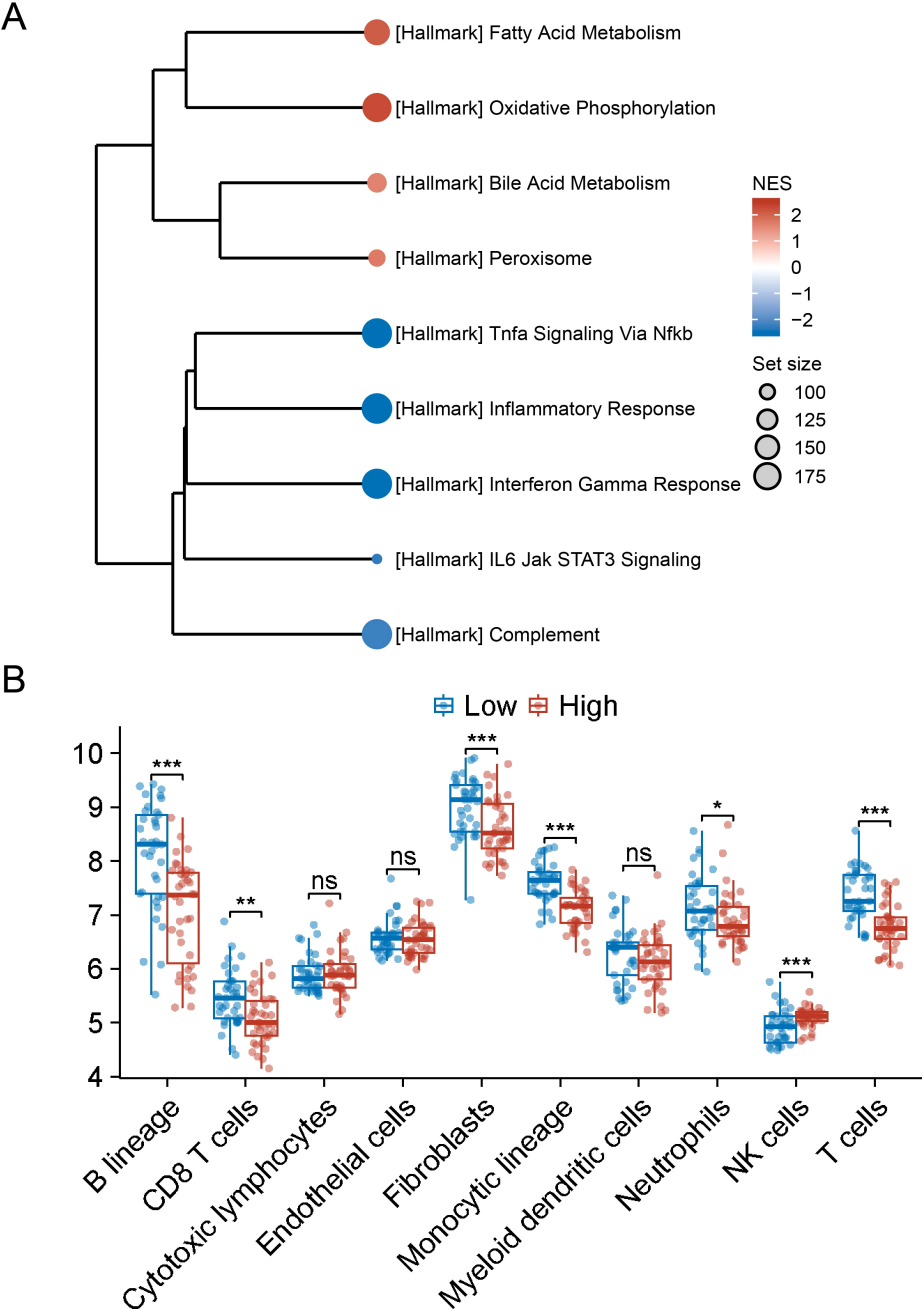
Innate inflammatory characteristics of GALNT12-defined subgroups in ulcerative colitis. **(A)** Hallmark gene set enrichment analysis comparing the GALNT12-high and GALNT12-low subgroups. Dot color represents the normalized enrichment score, and dot size indicates gene set size. **(B)** MCPcounter analysis comparing immune and stromal cell infiltration scores between the GALNT12-high and GALNT12-low subgroups. Statistical significance is indicated as ns, *P < 0.05, **P < 0.01, and ***P < 0.001.

After stratifying ulcerative colitis samples into GALNT12-high and GALNT12-low groups, Hallmark enrichment analysis revealed distinct biological states. The GALNT12-high group was enriched mainly in fatty acid metabolism, oxidative phosphorylation, bile acid metabolism, and peroxisome pathways, whereas the GALNT12-low group was enriched in TNFA signaling via NF-kB, inflammatory response, interferon gamma response, IL6-JAK-STAT3 signaling, and complement (**Figure 3A**). These findings suggest that lower GALNT12 expression is associated with a transcriptional context characterized by reduced epithelial metabolic programs and increased inflammatory activation.

MCPcounter analysis further showed that the GALNT12-low group had higher infiltration scores for B lineage cells, CD8 T cells, fibroblasts, monocyte lineage cells, neutrophils, and T cells (**Figure 3B**). In contrast, NK cells were relatively higher in the GALNT12-high group. No significant differences were observed for cytotoxic lymphocytes, endothelial cells, or myeloid dendritic cells. Together, these results indicate that reduced GALNT12 expression is associated with a more inflammatory tissue context enriched for innate-inflammatory and immune-interacting cell populations in UC.

### 3.4 GALNT12 was localized mainly to epithelial compartments at single-cell resolution

**Figure 4:**
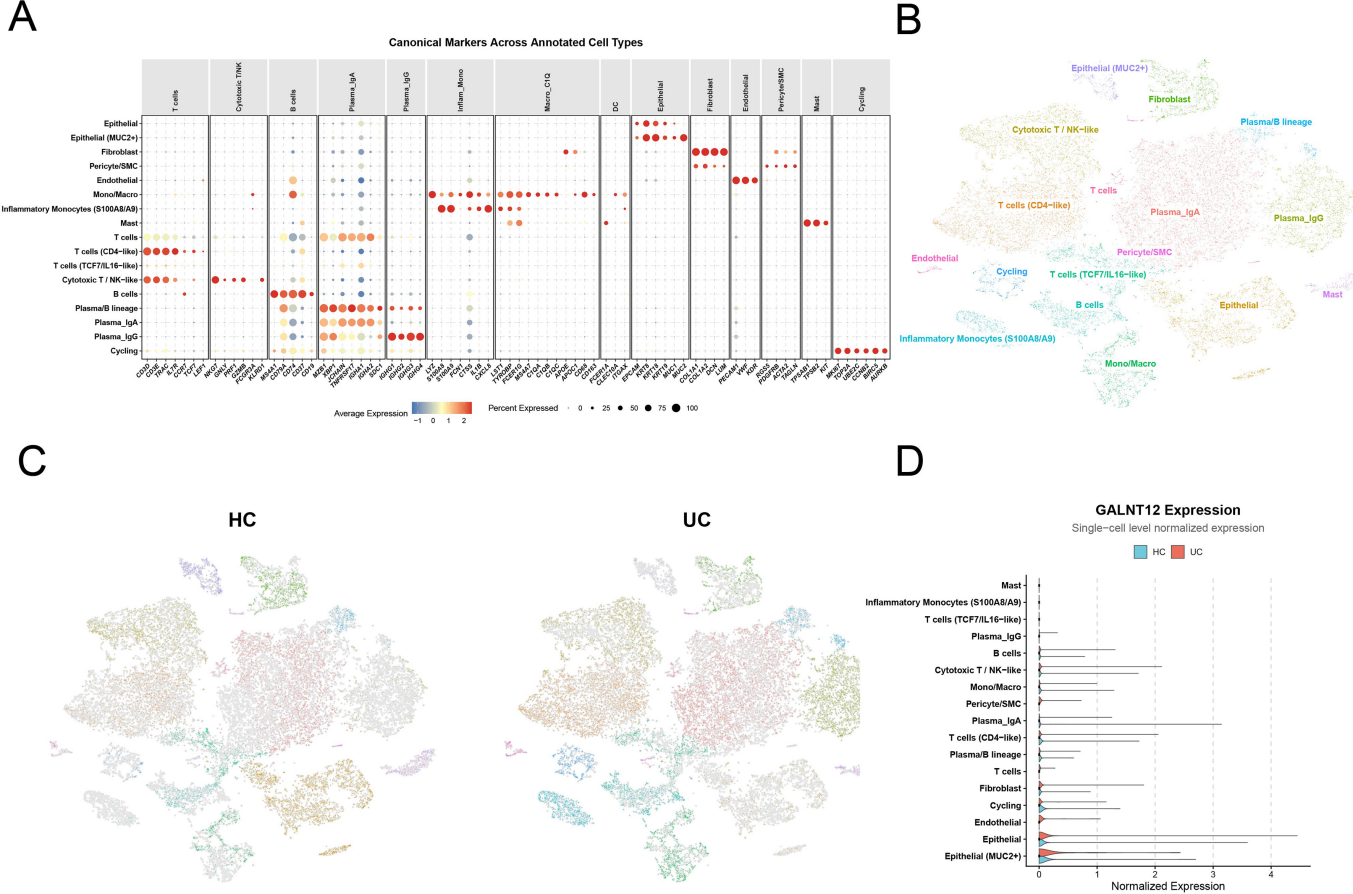
Single-cell transcriptional landscape and cellular distribution of GALNT12 in ulcerative colitis. **(A)** Dot plot showing canonical marker genes across annotated cell populations in the GSE214695 single-cell dataset. Dot size indicates the proportion of cells expressing each marker, and color indicates average expression. **(B)** UMAP plot showing the annotated major cell populations in the integrated single-cell dataset. **(C)** UMAP plots split by group, showing the cellular distributions in healthy control and ulcerative colitis samples. **(D)** Normalized GALNT12 expression across annotated cell populations. GALNT12 was concentrated in epithelial-related cell populations, particularly the epithelial MUC2-positive subset.

To define the cellular context of GALNT12, we analyzed the healthy control and ulcerative colitis samples from GSE214695. After integration and annotation, the dataset comprised epithelial cells, MUC2-positive epithelial cells, fibroblasts, endothelial cells, pericyte and smooth muscle cells, mono/macrophages, inflammatory monocytes, mast cells, B cells, plasma cell subsets, cycling cells, and several T-cell states (**Figures 4A–C**).

Normalized single-cell expression analysis showed that GALNT12 was concentrated in epithelial compartments, with the highest normalized expression observed in epithelial MUC2-positive cells and epithelial cells, whereas most stromal and immune populations showed low levels (**Figure 4D**). This cell-type distribution is consistent with the known role of GALNT12 in mucin-type O-glycosylation and supports an epithelial-centered interpretation of the bulk transcriptomic findings. Based on this pattern, the epithelial MUC2-positive subset was selected for downstream analyses.

### 3.5 GALNT12-positive MUC2-positive epithelial cells displayed broader epithelial-immune communication

**Figure 5:**
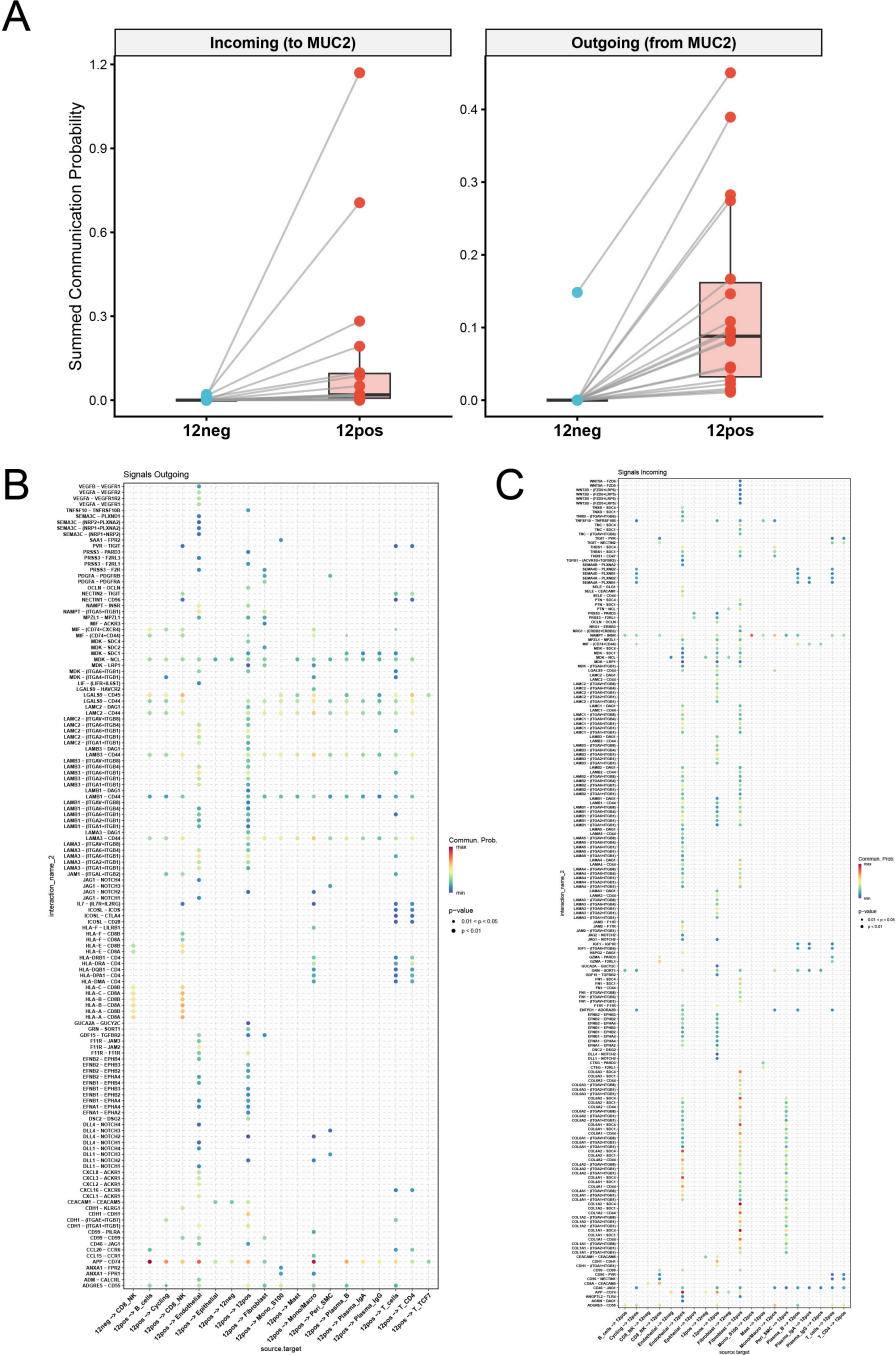
Cell-cell communication analysis of GALNT12-defined MUC2-positive epithelial subsets. **(A)** Paired comparison of summed communication probabilities for incoming and outgoing signaling in GALNT12-negative and GALNT12-positive epithelial MUC2-positive cells. **(B)** Bubble plot showing outgoing signaling interactions from GALNT12-negative and GALNT12-positive epithelial MUC2-positive cells to other populations. **(C)** Bubble plot showing incoming signaling interactions received by GALNT12-negative and GALNT12-positive epithelial MUC2-positive cells from other populations. For visualization, GALNT12-positive and GALNT12-negative epithelial MUC2-positive subsets were abbreviated as 12pos and 12neg, respectively.

Using the detectable-transcript-based 12pos/12neg stratification, GALNT12-positive cells in the ulcerative colitis epithelial MUC2-positive compartment showed higher summed communication probabilities than GALNT12-negative cells, particularly for outgoing signals. The summed communication probabilities were higher for both incoming and outgoing signals in 12pos cells, the difference being larger for outgoing signals (**Figure 5A**). A bubble plot analysis showed that 12pos cells had inferred interactions with a wider array of neighboring epithelial, stromal, and immune populations, whereas 12neg cells showed relatively sparse inferred engagement (**Figures 5B,C**). In agreement with this finding, circular network plots provided in supplementary data show restricted signaling of 12neg cells and broad outgoing connections of 12pos cells (**Supplementary Figure 1**). Combined, these results indicate that GALNT12 delineates a mucin-producing epithelial state that is more actively engaged in epithelial–immune crosstalk in the UC microenvironment.

### 3.6 GALNT12-associated epithelial programs linked secretory homeostasis to immune-facing responses

**Figure 6:**
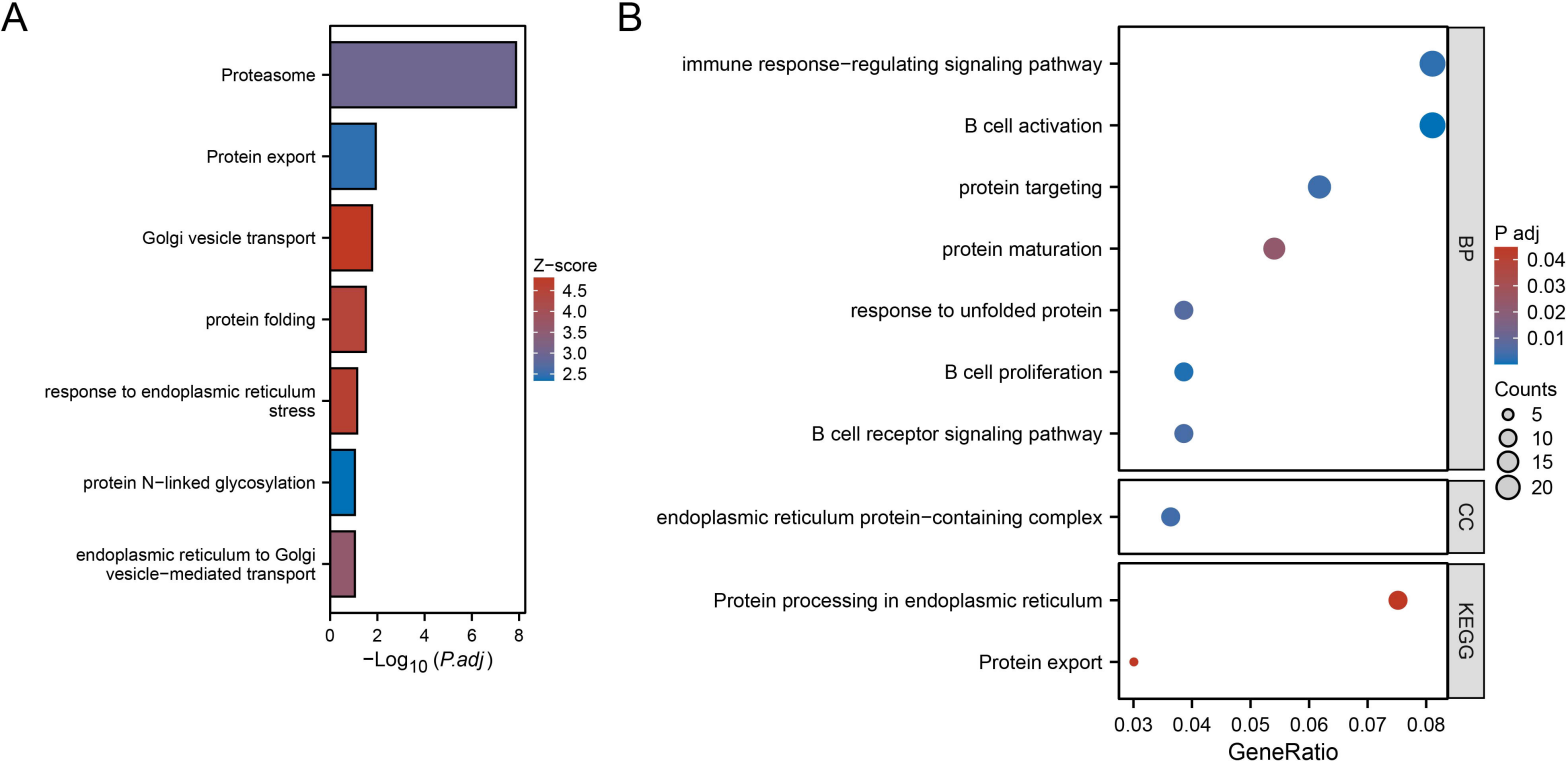
Functional analyses of GALNT12-associated programs in MUC2-positive epithelial cells. **(A)** Enrichment analysis based on differentially expressed genes between GALNT12-positive and GALNT12-negative cells within the ulcerative colitis epithelial MUC2-positive subset. Bar color indicates the enrichment Z-score. **(B)** Gene Ontology and KEGG enrichment analysis of genes perturbed after virtual knockout of GALNT12 in healthy control-derived GALNT12-positive epithelial MUC2-positive cells using scTenifoldKnk. Dot size indicates gene count, and dot color indicates adjusted P value.

To examine transcriptional programs associated with endogenous GALNT12 expression, we compared 12pos and 12neg cells within the ulcerative colitis epithelial MUC2-positive subset. Enrichment analysis of differentially expressed genes showed that GALNT12-positive cells were associated mainly with proteasome, protein export, Golgi vesicle transport, protein folding, response to endoplasmic reticulum stress, protein N-linked glycosylation, and endoplasmic reticulum to Golgi vesicle-mediated transport pathways (**Figure 6A**). These results link GALNT12 expression to secretory processing and intracellular proteostasis in epithelial cells.

We next performed virtual knockout of GALNT12 in healthy control-derived GALNT12-positive epithelial MUC2-positive cells to explore potential regulatory consequences of GALNT12 loss from a less inflamed epithelial baseline. Enrichment analysis of perturbed genes highlighted immune response-regulating signaling pathway, B cell activation, B cell receptor signaling pathway, B cell proliferation, protein targeting, protein maturation, and response to unfolded protein in the Gene Ontology categories, as well as protein processing in endoplasmic reticulum and protein export in KEGG analysis (**Figure 6B**). Taken together with the endogenous expression analysis, these findings suggest that GALNT12 is linked to epithelial secretory homeostasis while also influencing immune-facing epithelial programs.

### 3.7 Spatial transcriptomics identified a GALNT12-high mucin-associated niche with restrained inflammatory signaling

**Figure 7:**
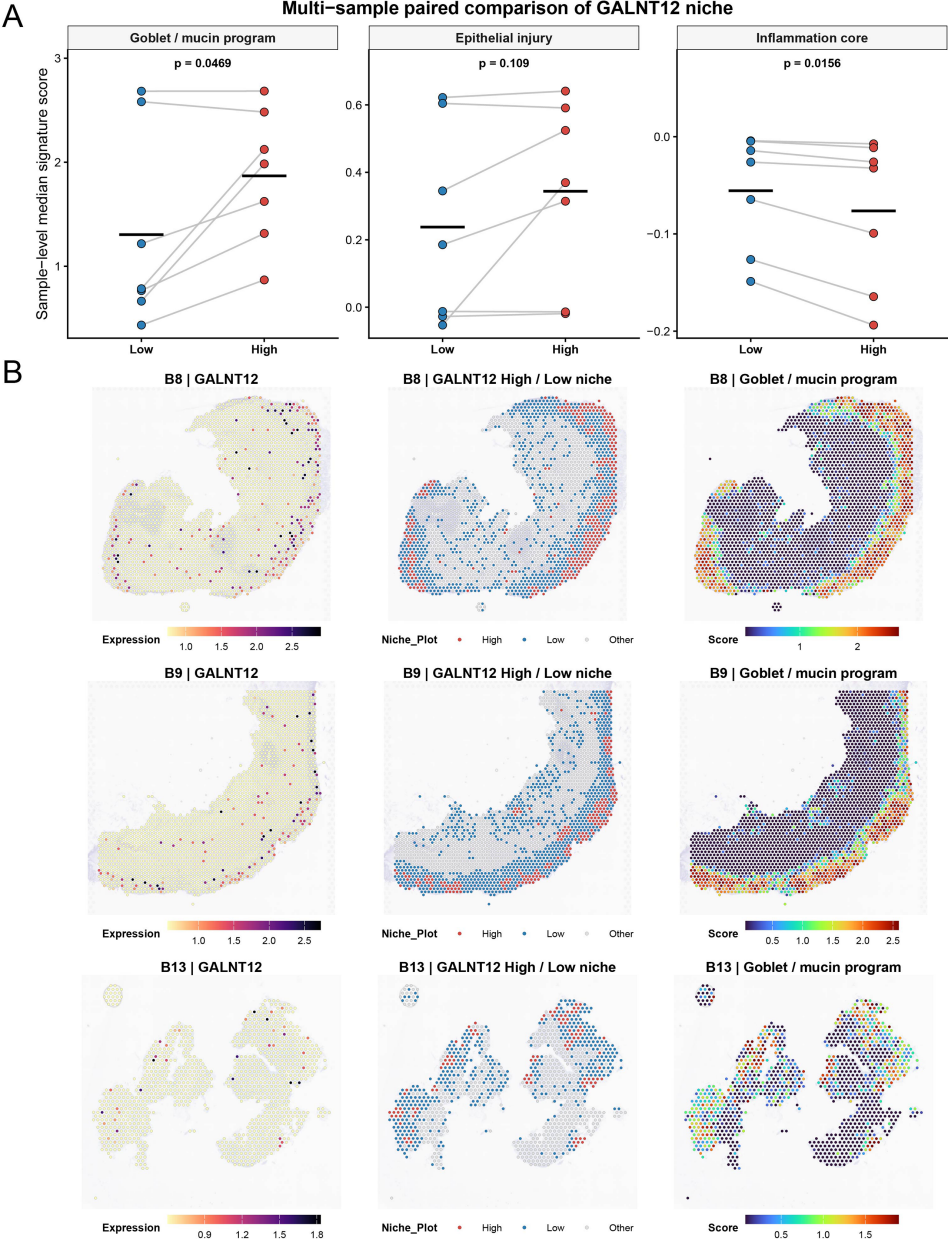
Spatial transcriptomic analysis identifies a GALNT12-high mucin-associated niche with restrained inflammatory signaling. **(A)** Multi-sample paired comparison of signature scores between GALNT12-high and GALNT12-low niches within MUC2-associated epithelial regions in the GSE189184 cohort. Sample-level median scores are shown for the goblet and mucin program, epithelial injury, and inflammation core signatures. **(B)** Representative spatial maps from B8, B9, and B13 showing GALNT12 expression, niche classification, and the spatial distribution of the goblet and mucin program.

To determine whether the GALNT12-associated epithelial state was spatially organized in tissue, we constructed a GALNT12-centered niche within MUC2-associated epithelial regions in GSE189184. Across seven analyzed samples, paired sample-level comparison showed that the goblet and mucin program score was higher in GALNT12-high niches than in GALNT12-low niches (P = 0.0469), whereas the inflammation core score was lower in GALNT12-high niches (P = 0.0156) (**Figure 7A**). The epithelial injury signature showed an upward trend in GALNT12-high niches but did not reach statistical significance (P = 0.109). Because this analysis was performed at spot-level resolution and based on seven samples, these results were interpreted as spatial associations.

Representative spatial maps from B8, B9, and B13 showed that GALNT12-high niches preferentially colocalized with regions exhibiting stronger goblet and mucin program scores (**Figure 7B** and **Supplementary Figure 2**). These spatial findings support the view that higher GALNT12 expression marks a mucin-associated epithelial niche with preserved secretory features and a relatively restrained inflammatory milieu.

### 3.8 GALNT12 knockdown enhanced TNF-α-induced inflammatory membrane injury and caspase-1/GSDMD-associated signaling *in vitro*

**Figure 8:**
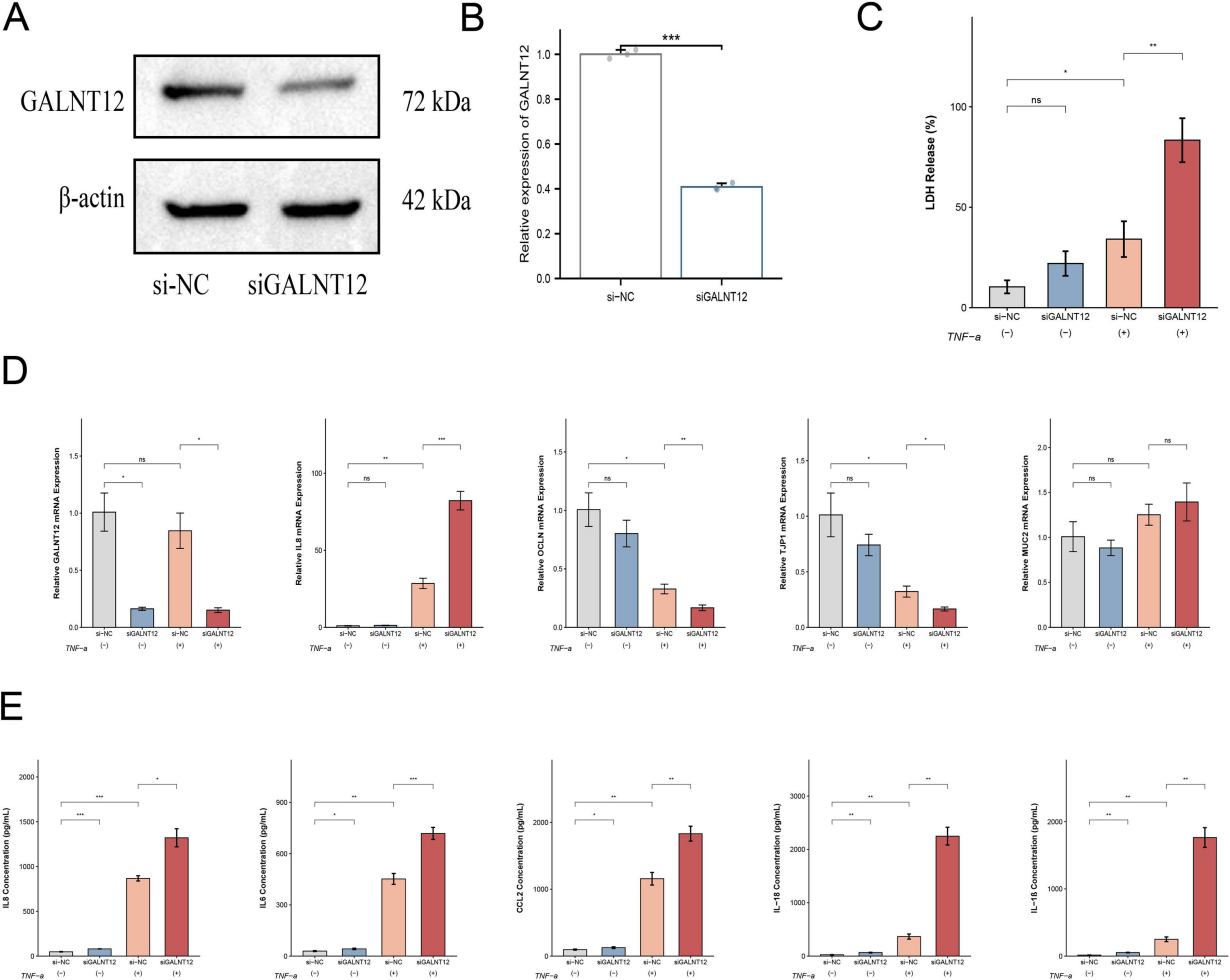
GALNT12 knockdown enhances TNF-α-induced inflammatory membrane injury and cytokine release in HT29-19A cells. **(A)** Representative Western blot showing GALNT12 protein expression in si-NC and siGALNT12 cells, with β-actin as the loading control. **(B)** Quantification of GALNT12 protein expression based on Western blot analysis. **(C)** LDH release assay showing inflammatory membrane injury in the four treatment groups. **(D)** RT-qPCR analysis of GALNT12, IL8, OCLN, TJP1, and MUC2 mRNA expression in HT29-19A cells. **(E)** ELISA measurement of IL-8, IL-6, CCL2, IL-1β, and IL-18 concentrations in culture supernatants. HT29-19A cells were divided into four groups: si-NC, siGALNT12, si-NC + TNF-α, and siGALNT12 + TNF-α. Data are presented as mean ± SD. *P < 0.05, **P < 0.01, ***P < 0.001; ns, not significant. The original uncropped full-scan images for the western blots are provided as Supplementary Information (**Supplementary Figure 3**).

To verify the efficiency of GALNT12 silencing, Western blotting and densitometric quantification were first performed. GALNT12 protein expression was markedly reduced in siGALNT12-transfected HT29-19A cells compared with si-NC controls (**Figures 8A, B**). This knockdown effect was further confirmed at the transcriptional level by RT-qPCR, which showed a consistent decrease in GALNT12 mRNA in the siGALNT12-treated groups (**Figure 8D**).

We next examined whether GALNT12 deficiency affected epithelial membrane stability under TNF-α-induced inflammatory stress. LDH release remained low in the si-NC group and showed only a modest increase after GALNT12 knockdown alone. TNF-α stimulation increased LDH release, and the highest LDH release was observed in the siGALNT12 plus TNF-α group (**Figure 8C**). These results indicate that GALNT12 deficiency sensitizes epithelial cells to TNF-α-induced membrane injury.

We then assessed inflammatory mediator production and barrier-related gene expression. TNF-α stimulation markedly increased IL8 mRNA expression, and this response was further amplified by GALNT12 knockdown. In contrast, the tight-junction genes OCLN and TJP1 were downregulated after TNF-α stimulation and declined further in the siGALNT12 plus TNF-α group (**Figure 8D**). MUC2 mRNA did not show a significant suppressive trend, suggesting that GALNT12 may influence mucin-associated epithelial function primarily through post-transcriptional or glycosylation-related mechanisms rather than direct transcriptional regulation of MUC2.

Consistent with the transcriptional changes, ELISA showed that TNF-α robustly induced the secretion of IL-8, IL-6, and CCL2, and these responses were further enhanced by GALNT12 knockdown (**Figure 8E**). IL-1β and IL-18 secretion showed a similar pattern, with the strongest increase observed in the siGALNT12 plus TNF-α group (**Figure 8E**). Together, the **Figure 8** data indicate that GALNT12 deficiency aggravates TNF-α-induced epithelial membrane injury, barrier-related gene suppression, and inflammatory mediator output.

**Figure 9:**
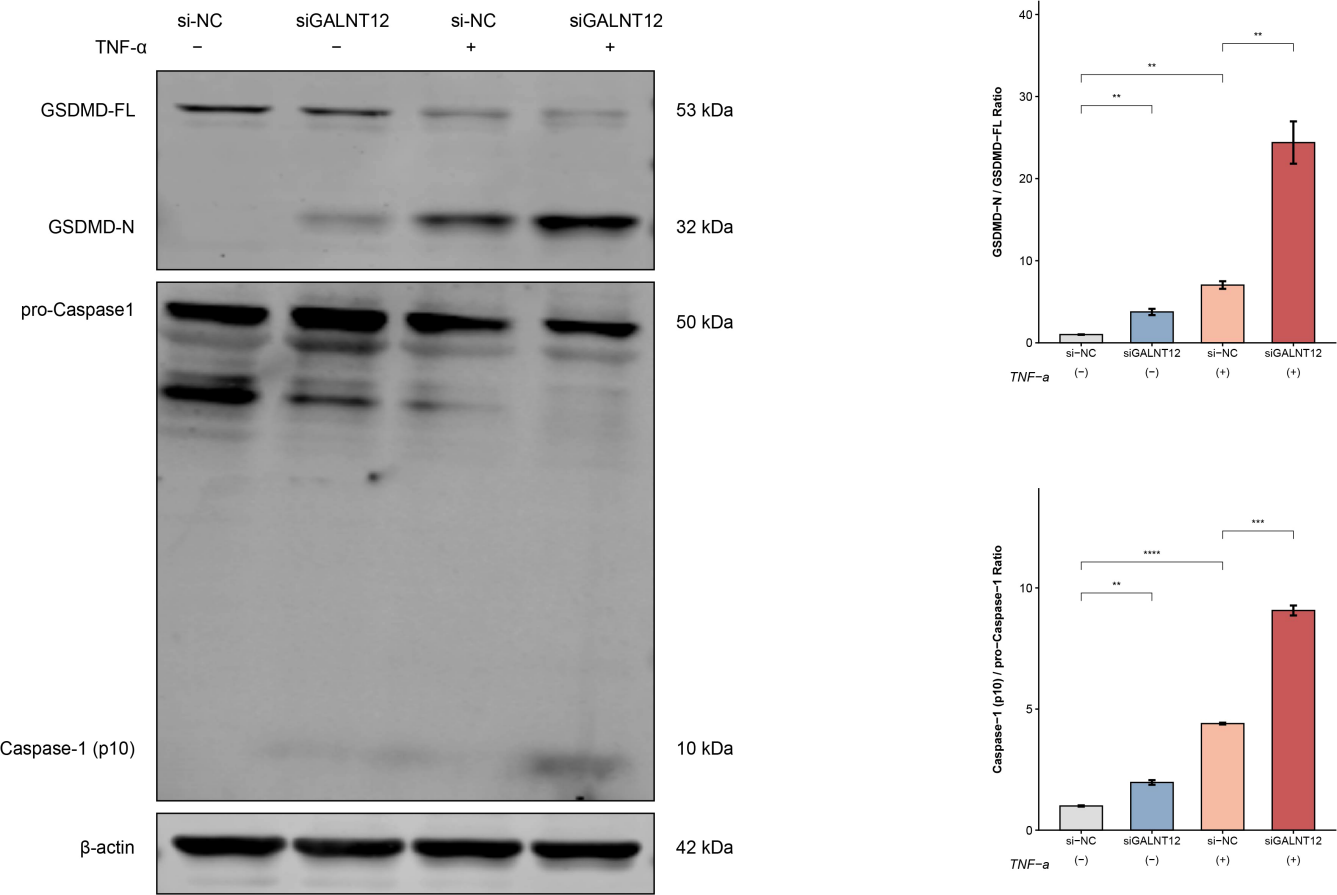
GALNT12 knockdown enhances TNF-α-induced caspase-1/GSDMD-associated signaling in HT29-19A cells. Data are presented as mean ± SD. *P < 0.05, **P < 0.01, ***P < 0.001, ****P < 0.0001; ns, not significant. Original uncropped full-scan images corresponding to the Western blots are provided as **Supplementary Figure 4**.

To further determine whether the increased LDH release and IL-1β/IL-18 output were accompanied by caspase-1/GSDMD-associated signaling, we performed additional Western blotting for caspase-1 and GSDMD. Caspase-1 processing, assessed by cleaved caspase-1 p10 and the relative p10/pro-caspase-1 ratio, was increased after TNF-α stimulation and was most prominent in the si-GALNT12 plus TNF-α group (**Figure 9**). GSDMD cleavage showed a similar pattern. GSDMD-N formation and the relative GSDMD-N/full-length GSDMD ratio were increased under TNF-α stress and were further enhanced by GALNT12 knockdown, with the strongest signal observed in the siGALNT12 plus TNF-α group (**Figure 9**).

These findings provide additional support for a caspase-1/GSDMD-associated inflammatory injury phenotype in GALNT12-deficient epithelial cells under cytokine stress. However, these data do not by themselves prove complete pyroptotic execution at the single-cell level.

## 4 Discussion

In this study, we integrated bulk, single-cell, and spatial transcriptomic analyses with *in vitro* perturbation to examine how mucin-type O-glycosylation-related biology intersects with inflammatory epithelial injury in ulcerative colitis. Several findings converged. First, the bulk analyses identified coordinated dysregulation of a compact mucin-type O-glycosylation gene set enriched for glycosyltransferase functions, O-glycan biosynthesis, and Golgi-associated secretory processes. Second, an integrative prioritization framework consistently highlighted GALNT12 as the leading epithelial candidate. Third, across transcriptomic, spatial, and functional layers, reduced GALNT12 expression was associated with an inflammatory epithelial state and stronger immune-interacting tissue features, whereas GALNT12 knockdown increased susceptibility to TNF-α-induced membrane injury, inflammatory cytokine release, and caspase-1/GSDMD-associated signaling.

The bulk transcriptomic results are consistent with the idea that UC involves remodeling rather than uniform loss of mucus-associated glycosylation machinery. Alongside reduced CLCA1, B3GNT8, GALNT12, and MUC20, we observed relatively higher expression of several enzymes involved in sialylation, fucosylation, and related glycan remodeling, as well as XBP1 and several mucin-associated genes. Prior work has shown that active UC is accompanied by altered MUC2 O-glycan composition and impaired mucus barrier architecture (5–7). Experimental and translational studies also indicate that mucin glycosylation can affect microbial interactions and inflammatory signaling (28, 29). Our results extend this literature by suggesting that the UC epithelium undergoes coordinated reconfiguration of mucin-related enzymes and secretory programs that is linked to inflammatory amplification at the barrier-immune interface.

GALNT12 was notable because it remained important across several complementary prioritization strategies. GALNT12 belongs to the GalNAc-transferase family that initiates mucin-type O-glycosylation, a process with unusually high regulatory diversity in epithelial tissues (8). GALNT12 is expressed mainly in digestive organs and has activity toward mucin-derived peptide substrates (9). Although GALNT12 has been discussed mainly in the context of colorectal tumor susceptibility and enzyme structure-function relationships (10–12), its role in inflammatory intestinal disease has been much less defined. The present data suggest that GALNT12 is relevant to UC not only as a marker of epithelial differentiation but also as an indicator of epithelial functional state.

The subgroup analysis further placed GALNT12 in an inflammatory tissue context. Lower GALNT12 expression was associated with TNFA signaling via NF-kB, interferon gamma response, IL6-JAK-STAT3 signaling, and complement, together with higher estimated abundance of B lineage cells, monocyte lineage cells, neutrophils, fibroblasts, CD8 T cells, and total T cells. These patterns fit the broader view from single-cell studies showing that UC involves epithelial rewiring together with marked stromal, myeloid, and adaptive immune remodeling (13, 15–17). In this setting, GALNT12-low tissue may reflect mucosa in which epithelial secretory competence has been eroded and inflammatory crosstalk has intensified, especially in relation to innate-inflammatory recruitment and activation.

We used single-cell and spatial transcriptomic analyses to obtain finer localization of these processes. The localization to epithelial cells and MUC2-positive epithelial cells has the expected biological significance, as goblet cells and mucin-secreting cells are integral to the process of O-glycosylated mucus production (4, 14). Based on our findings, cells with higher GALNT12 expression had more extensive networks of incoming and outgoing communication in this compartment than cells with lower GALNT12 expression, suggesting that GALNT12 marks an epithelial state with enhanced tissue crosstalk. According to their transcriptomic profile, these GALNT12-positive cells were enriched for pathways associated with the proteasome, protein folding, endoplasmic reticulum stress, and Golgi function. Secretory epithelial cells are required to integrate mucin synthesis with folding, glycosylation, and quality control. Thus, patterns of this type are significant. According to our virtual knockout simulation, we found that loss of GALNT12 is destabilizing not only to secretory homeostasis but also to immune-response-regulating programs, indicating that epithelial glycosylation status shapes immune-oriented epithelial functions. Our research benefited from the incorporation of spatial transcriptomic data. Niches with high GALNT12 expression found in MUC2-associated epithelial regions carried increased goblet and mucin program scores and lower inflammation core scores. These spatial data suggest that GALNT12 does not act merely as a passenger marker but identifies a relatively preserved mucin-associated epithelial niche. Despite the upward trend seen in the epithelial injury signature of the niches, we note a lack of significance, which may be due to sample size. The coexistence of protective and regenerative responses will require further validation in future studies.

*In vitro* experiments provided additional mechanistic support. In our updated functional analysis, GALNT12 silencing further exacerbated TNF-α-induced LDH release, enhanced the secretion of IL-8, IL-6, CCL2, IL-1β, and IL-18, while OCLN and TJP1 decreased further under inflammatory stress. The results presented indicate that GALNT12 deficiency sensitizes epithelial cells to inflammatory membrane injury and intensifies a cytokine program enriched for innate inflammatory mediators. Our data support a pyroptosis-associated phenotype under cytokine stress, as the increased release of LDH along with increased levels of IL-1β and IL-18 is consistent with lytic inflammatory cell damage.

This study has several limitations. First, public transcriptomic datasets generated on different platforms may introduce unavoidable technical variation. Second, the GALNT12-positive/GALNT12-negative classification in scRNA-seq was based on detectable transcript counts and may be influenced by transcript sparsity and dropout. Third, CellChat and scTenifoldKnk are inference-based approaches and should be regarded as hypothesis-generating rather than direct biochemical validation. Fourth, the spatial transcriptomic analysis was based on seven samples and spot-level resolution; therefore, GALNT12-high niches may partly reflect regions enriched for intact mucin-producing epithelial cells and relatively fewer inflammatory cells. Fifth, the *in vitro* validation was performed in HT29-19A cells, which are useful mucin-secretory epithelial cells but remain cancer-derived and cannot fully substitute for primary colonic epithelial cells or UC patient-derived organoids. Finally, although the added Western blot data support caspase-1/GSDMD-associated signaling, this study did not include GALNT12 rescue/overexpression experiments, direct MUC2 glycoform or O-glycan structural analysis, primary epithelial organoid validation, or *in vivo* validation. These limitations should be addressed in future studies.

## Data Availability

Public transcriptomic datasets analyzed in this study are available in the Gene Expression Omnibus under accession numbers GSE107499, GSE47908, GSE87466, GSE214695, and GSE189184. Additional code and source data are available from the corresponding authors upon reasonable request.

## Supplementary material

Supplementary Figure 1 Circular network visualization of outgoing signaling from GALNT12-defined epithelial MUC2-positive subsets. Left, outgoing communication network of the 12neg subgroup; right, outgoing communication network of the 12pos subgroup. The plots illustrate the relative breadth and strength of outgoing intercellular communication from each subgroup to surrounding cell populations.

Supplementary Figure 2 Spatial distribution of GALNT12 and mucin-associated niches across all analyzed colonic biopsy sections. Spatial transcriptomic panels for all seven ulcerative colitis tissue slices (B4, B5, B8, B9, B12, B13, and C2). For each slice, the H&E staining, spatial expression of GALNT12, and the defined mucin-associated epithelial niches are displayed to support the consistency of the spatial findings.

Supplementary Figure 3 Original full-scan images of the western blots. The uncropped blot images corresponding to the experiments shown in **Figure 8A** are presented.

Supplementary Figure 4 Original full-scan images of the western blots. The uncropped blot images corresponding to the experiments shown in **Figure 9** are presented.

## References

[1] Ordás I Eckmann L Talamini M Baumgart DC Sandborn WJ . Ulcerative colitis. Lancet. (2012) 380:1606–19. doi:10.1016/S0140-6736(12)60150-0. PMID: 22914296

[2] Dunleavy KA Raffals LE Camilleri M . Intestinal barrier dysfunction in inflammatory bowel disease: underpinning pathogenesis and therapeutics. Dig Dis Sci. (2023) 68:4306–20. doi:10.1007/s10620-023-08122-w. PMID: 37773554 PMC10798146

[3] Wang M Shi J Yu C Zhang X Xu G Xu Z . Emerging strategy towards mucosal healing in inflammatory bowel disease: what the future holds? Front Immunol. (2023) 14:1298186. doi:10.3389/fimmu.2023.1298186. PMID: 38155971 PMC10752988

[4] Johansson MEV . Mucus layers in inflammatory bowel disease. Inflammation Bowel Dis. (2014) 20:2124–31. doi:10.1097/MIB.0000000000000117. PMID: 25025717

[5] Johansson MEV Gustafsson JK Holmen-Larsson J Jabbar KS Xia L Xu H . Bacteria penetrate the normally impenetrable inner colon mucus layer in both murine colitis models and patients with ulcerative colitis. Gut. (2014) 63:281–91. doi:10.1136/gutjnl-2012-303207. PMID: 23426893 PMC3740207

[6] van der Post S Jabbar KS Birchenough G Arike L Akhtar N Sjövall H . Structural weakening of the colonic mucus barrier is an early event in ulcerative colitis pathogenesis. Gut. (2019) 68:2142–51. doi:10.1136/gutjnl-2018-317571. PMID: 30914450 PMC6872445

[7] Holmn Larsson JM Karlsson H Gråberg Crespo J Johansson MEV Eklund L Sjövall H . Altered O-glycosylation profile of MUC2 mucin occurs in active ulcerative colitis and is associated with increased inflammation. Inflammation Bowel Dis. (2011) 17:2299–307. doi:10.1002/ibd.21625. PMID: 21290483

[8] Bennett EP Mandel U Clausen H Gerken TA Fritz TA Tabak LA . Control of mucin-type O-glycosylation: a classification of the polypeptide GalNAc-transferase gene family. Glycobiology. (2012) 22:736–56. doi:10.1093/glycob/cwr182. PMID: 22183981 PMC3409716

[9] Guo JM Zhang Y Cheng L Iwasaki H Wang H Kubota T . Molecular cloning and characterization of a novel member of the UDP-GalNAc:polypeptide N-acetylgalactosaminyltransferase family, pp-GalNAc-T12. FEBS Lett. (2002) 524:211–8. doi:10.1016/S0014-5793(02)03007-7. PMID: 12135769

[10] Guda K Moinova H He J Jamison O Ravi L Natale L . Inactivating germ-line and somatic mutations in polypeptide N-acetylgalactosaminyltransferase 12 in human colon cancers. Proc Natl Acad Sci USA. (2009) 106:12921–5. doi:10.1073/pnas.0901454106. PMID: 19617566 PMC2722285

[11] Evans DR Venkitachalam S Revoredo L Dohey AT Clarke E Pennell JJ . Evidence for GALNT12 as a moderate penetrance gene for colorectal cancer. Hum Mutat. (2018) 39:1092–101. doi:10.1002/humu.23549. PMID: 29749045 PMC6043371

[12] Fernandez AJ Daniel EJP Mahajan SP Gray JJ Gerken TA Tabak LA . The structure of the colorectal cancer-associated enzyme GalNAc-T12 reveals how nonconserved residues dictate its function. Proc Natl Acad Sci USA. (2019) 116:20404–10. doi:10.1073/pnas.1902211116. PMID: 31548401 PMC6789641

[13] Smillie CS Biton M Ordovas-Montanes J Sullivan KM Burgin G Graham DB . Intra- and inter-cellular rewiring of the human colon during ulcerative colitis. Cell. (2019) 178:714–730.e22. doi:10.1016/j.cell.2019.06.029. PMID: 31348891 PMC6662628

[14] Parikh K Antanaviciute A Fawkner-Corbett D Jagielowicz M Aulicino A Lagerholm C . Colonic epithelial cell diversity in health and inflammatory bowel disease. Nature. (2019) 567:49–55. doi:10.1038/s41586-019-0992-y. PMID: 30814735

[15] Kinchen J Chen HH Parikh K Antanaviciute A Jagielowicz M Fawkner-Corbett D . Structural remodeling of the human colonic mesenchyme in inflammatory bowel disease. Cell. (2018) 175:372–386.e17. doi:10.1016/j.cell.2018.08.067. PMID: 30270042 PMC6176871

[16] Boland BS He Z Tsai MS Olvera JG Omilusik KD Duong HG . Heterogeneity and clonal relationships of adaptive immune cells in ulcerative colitis revealed by single-cell analyses. Sci Immunol. (2020) 5:eabb4432. doi:10.1126/sciimmunol.abb4432. PMID: 32826341 PMC7733868

[17] Garrido-Trigo A Corraliza AM Veny M Dotti I Melón-Ardanaz E Rill A . Macrophage and neutrophil heterogeneity at single-cell spatial resolution in human inflammatory bowel disease. Nat Commun. (2023) 14:4506. doi:10.1038/s41467-023-40156-6. PMID: 37495570 PMC10372067

[18] Ritchie ME Phipson B Wu D Hu Y Law CW Shi W . limma powers differential expression analyses for RNA-sequencing and microarray studies. Nucleic Acids Res. (2015) 43:e47. doi:10.1093/nar/gkv007. PMID: 25605792 PMC4402510

[19] Szklarczyk D Kirsch R Koutrouli M Nastou K Mehryary F Hachilif R . The STRING database in 2023: protein-protein association networks and functional enrichment analyses for any sequenced genome of interest. Nucleic Acids Res. (2023) 51:D638–46. doi:10.1093/nar/gkac1000. PMID: 36370105 PMC9825434

[20] Wu T Hu E Xu S Chen M Guo P Dai Z . clusterProfiler 4.0: a universal enrichment tool for interpreting omics data. Innovation (Camb). (2021) 2:100141. doi:10.1016/j.xinn.2021.100141. PMID: 34557778 PMC8454663

[21] Yu G Li F Qin Y Bo X Wu Y Wang S . GOSemSim: an R package for measuring semantic similarity among GO terms and gene products. Bioinformatics. (2010) 26:976–8. doi:10.1093/bioinformatics/btq064. PMID: 20179076

[22] Lundberg SM Lee SI . A unified approach to interpreting model predictions. Adv Neural Inf Process Syst. (2017) 30:4765–74. doi: 10.5555/3295222.3295230

[23] Liberzon A Birger C Thorvaldsdóttir H Ghandi M Mesirov JP Tamayo P . The Molecular Signatures Database hallmark gene set collection. Cell Syst. (2015) 1:417–25. doi:10.1016/j.cels.2015.12.004. PMID: 26771021 PMC4707969

[24] Becht E Giraldo NA Lacroix L Buttard B Elarouci N Petitprez F . Estimating the population abundance of tissue-infiltrating immune and stromal cell populations using gene expression. Genome Biol. (2016) 17:218. doi:10.1186/s13059-016-1070-5. PMID: 27765066 PMC5073889

[25] Hao Y Hao S Andersen-Nissen E Mauck W Zheng S Butler A . Integrated analysis of multimodal single-cell data. Cell. (2021) 184:3573–3587.e29. doi:10.1016/j.cell.2021.04.048. PMID: 34062119 PMC8238499

[26] Jin S Guerrero-Juarez CF Zhang L Chang I Ramos R Kuan CH . Inference and analysis of cell-cell communication using CellChat. Nat Commun. (2021) 12:1088. doi:10.1038/s41467-021-21246-9. PMID: 33597522 PMC7889871

[27] Osorio D Zhong Y Li G Xu Q Yang Y Tian Y . scTenifoldKnk: an efficient virtual knockout tool for gene function predictions via single-cell gene regulatory network perturbation. Patterns (N Y). (2022) 3:100434. doi:10.1016/j.patter.2022.100434. PMID: 35510185 PMC9058914

[28] Arike L Holmén-Larsson J Hansson GC . Intestinal Muc2 mucin O-glycosylation is affected by microbiota and regulated by differential expression of glycosyltransferases. Glycobiology. (2017) 27:318–28. doi:10.1093/glycob/cww134. PMID: 28122822 PMC5444243

[29] Wei J Chen C Feng J Zhou S Feng X Yang Z . Muc2 mucin O-glycosylation interacts with enteropathogenic Escherichia coli to influence the development of ulcerative colitis based on the NF-kB signaling pathway. J Transl Med. (2023) 21:793. doi:10.1186/s12967-023-04687-2. PMID: 37940996 PMC10631195

