# Supplementary material for: Multi-layer transcriptomic analyses identify a mucin-associated epithelial program linked to innate inflammatory injury in ulcerative colitis
Source: Front Immunol. 2026 Jun 3;17:1846672. doi: 10.3389/fimmu.2026.1846672 (PMC13271950; doi:10.3389/fimmu.2026.1846672)
Supplement: Supplementary Figure 1 — Circular network visualization of outgoing signaling from GALNT12-defined epithelial MUC2-positive subsets. Left, outgoing communication network of the 12neg subgroup; right, outgoing communication network of the 12pos subgroup. The plots illustrate the relative breadth and strength of outgoing intercellular communication from each subgroup to surrounding cell populations. [file DataSheet1.pdf]

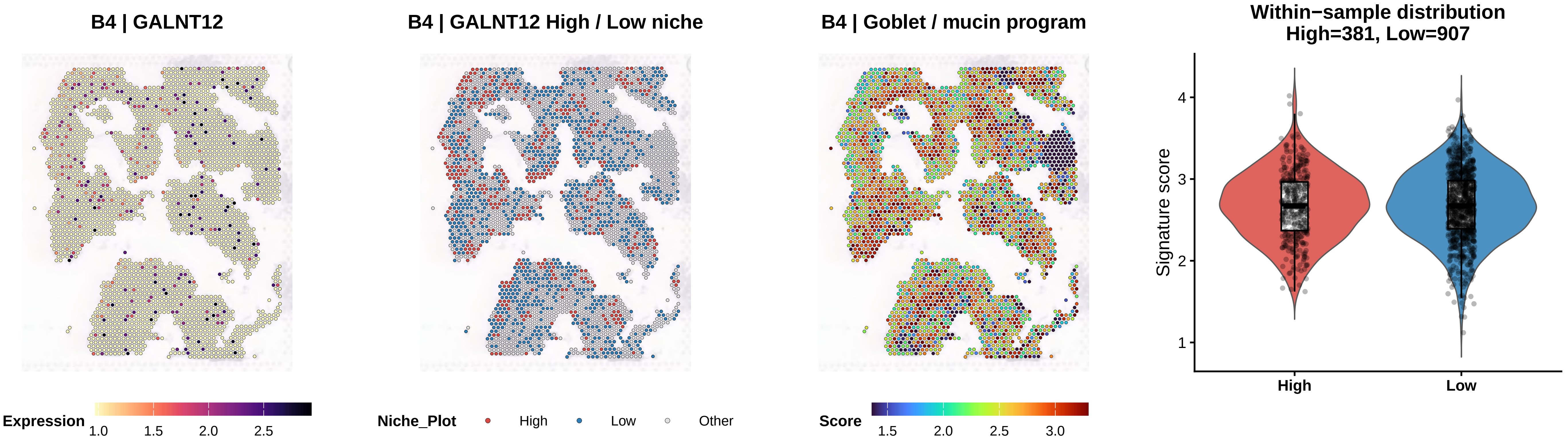

B4 | GALNT12
Expression
1.0
1.5
2.0
2.5
B4 | GALNT12 High / Low niche
Niche_Plot
High
Low
Other
B4 | Goblet / mucin program
Score
1.5
2.0
2.5
3.0
Within−sample distribution
High=381, Low=907
Signature score
1
2
3
4
High
Low

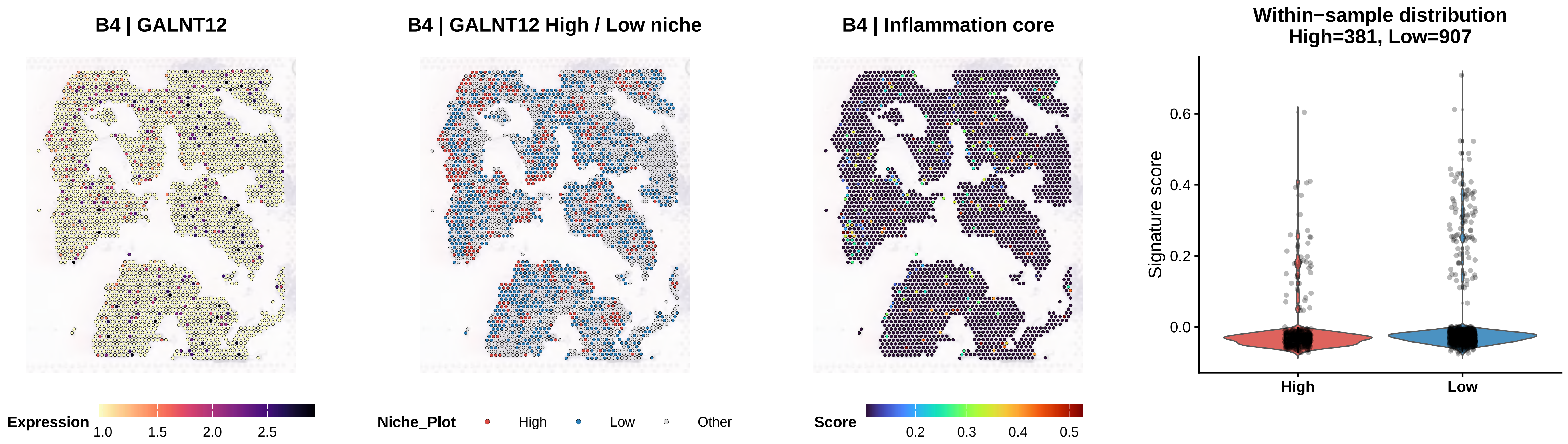
B4 | GALNT12
Expression
1.0
1.5
2.0
2.5
B4 | GALNT12 High / Low niche
Niche_Plot
High
Low
Other
B4 | Inflammation core
Score
0.2
0.3
0.4
0.5
Within−sample distribution
High=381, Low=907
Signature score
0.0
0.2
0.4
0.6
High
Low

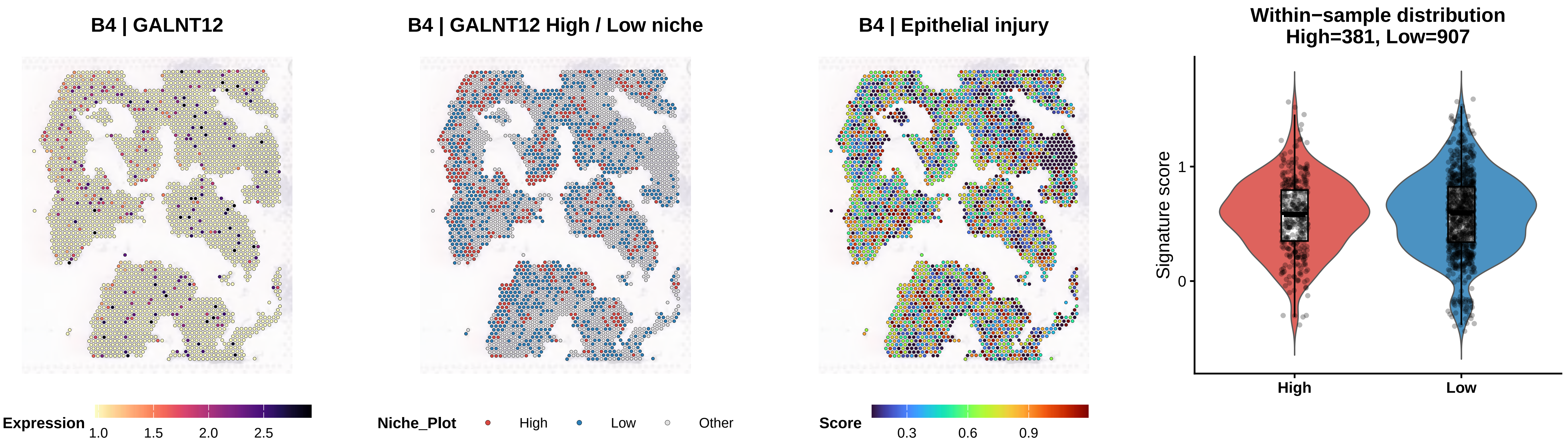
B4 | GALNT12
B4 | GALNT12 High / Low niche
B4 | Epithelial injury
Within−sample distribution
High=381, Low=907
Expression
1.0
1.5
2.0
2.5
Niche_Plot
High
Low
Other
Score
0.3
0.6
0.9
Signature score
1
0
High
Low

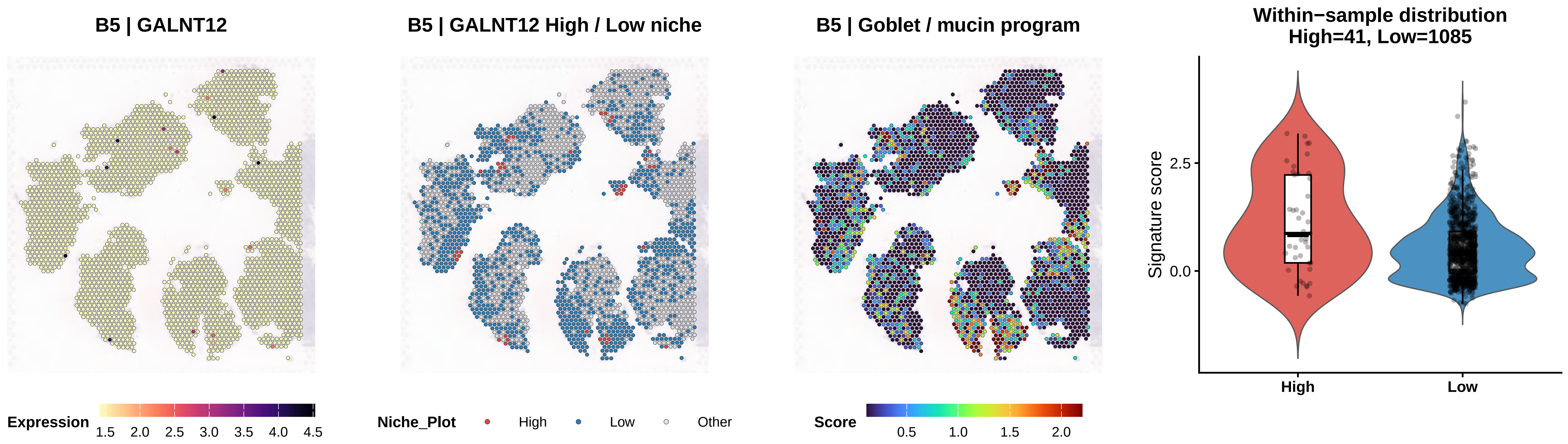
B5 | GALNT12
Expression
1.5
2.0
2.5
3.0
3.5
4.0
4.5
B5 | GALNT12 High / Low niche
Niche_Plot
High
Low
Other
B5 | Goblet / mucin program
Score
0.5
1.0
1.5
2.0
Within−sample distribution
High=41, Low=1085
Signature score
2.5
0.0
High
Low

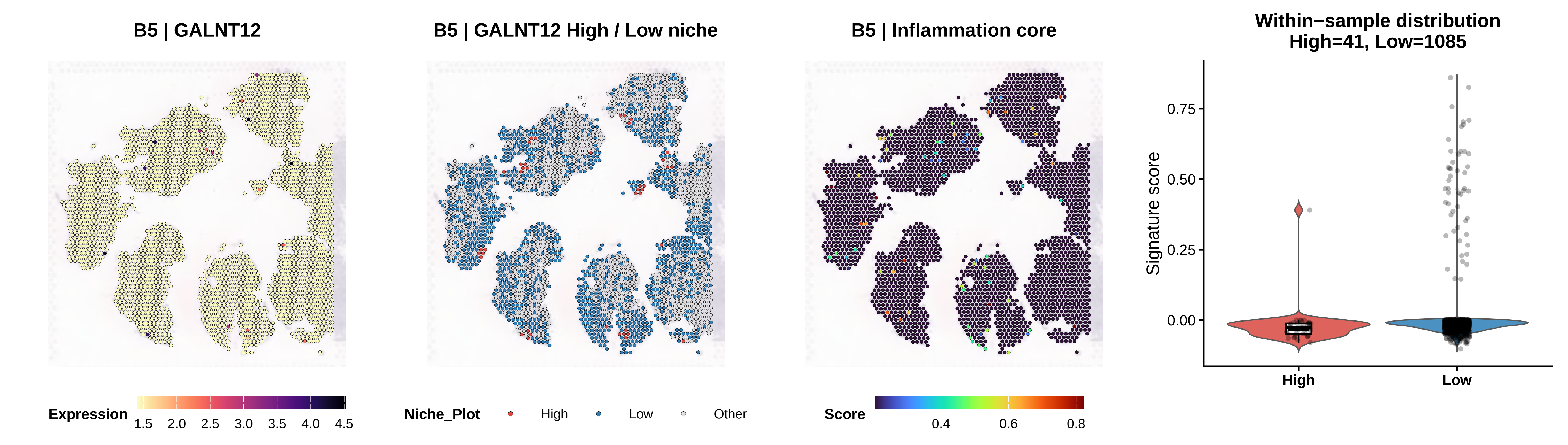
B5 | GALNT12
B5 | GALNT12 High / Low niche
B5 | Inflammation core
Within−sample distribution
High=41, Low=1085
Signature score
0.75
0.50
0.25
0.00
High
Low
Expression
1.5
2.0
2.5
3.0
3.5
4.0
4.5
Niche_Plot
High
Low
Other
Score
0.4
0.6
0.8

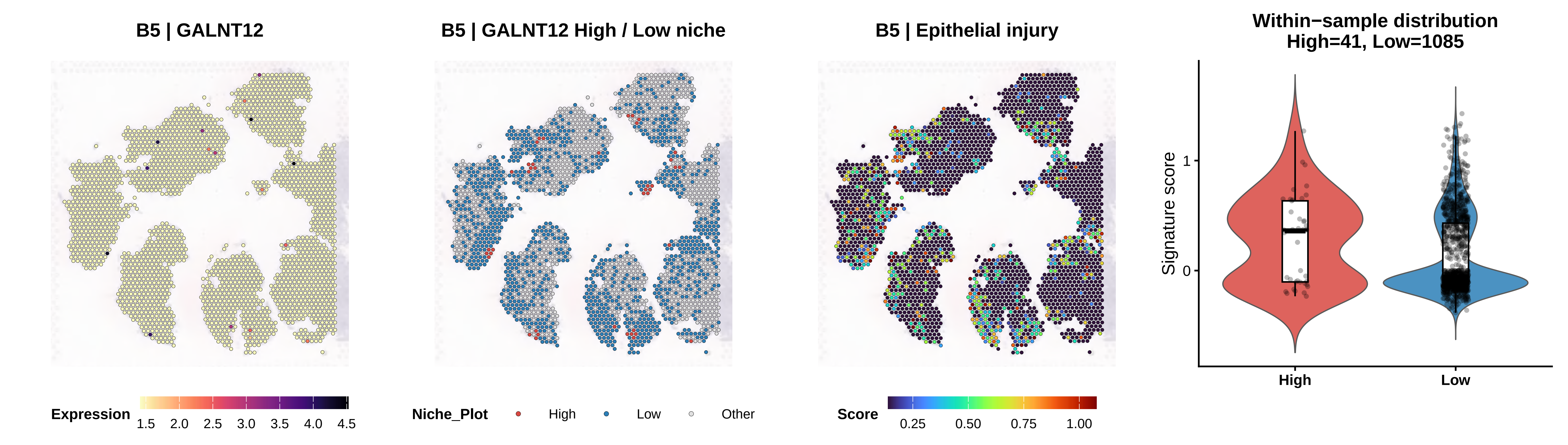

B5 | GALNT12
B5 | GALNT12 High / Low niche
B5 | Epithelial injury
Within−sample distribution
High=41, Low=1085
Signature score
1
0
High
Low
Expression
1.5
2.0
2.5
3.0
3.5
4.0
4.5
Niche_Plot
High
Low
Other
Score
0.25
0.50
0.75
1.00

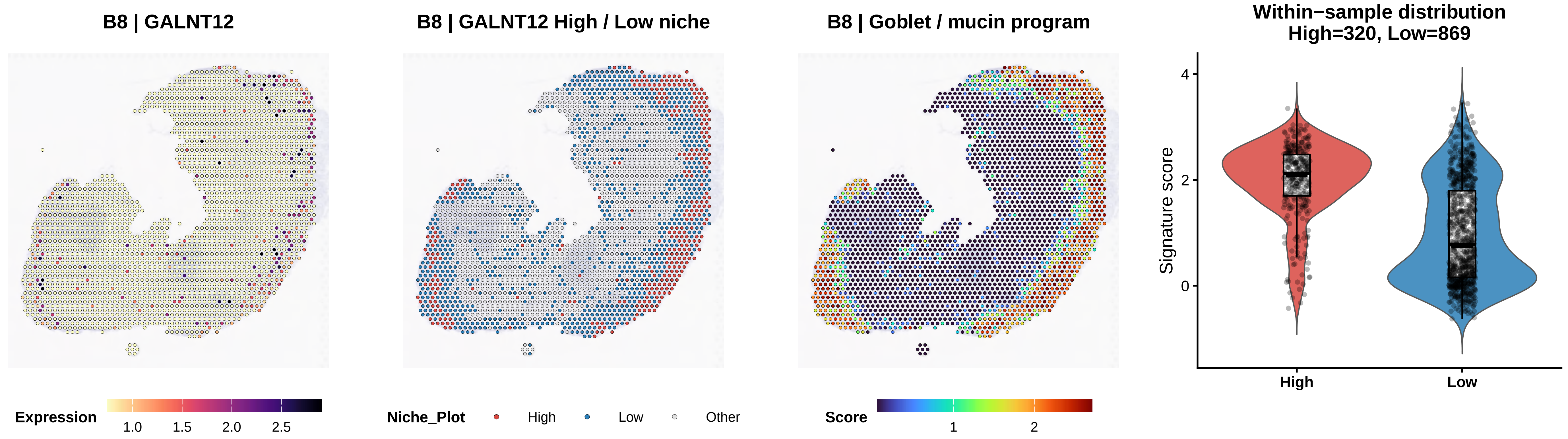

B8 | GALNT12
Expression
1.0
1.5
2.0
2.5
B8 | GALNT12 High / Low niche
Niche_Plot
High
Low
Other
B8 | Goblet / mucin program
Score
1
2
Within−sample distribution
High=320, Low=869
Signature score
4
2
0
High
Low

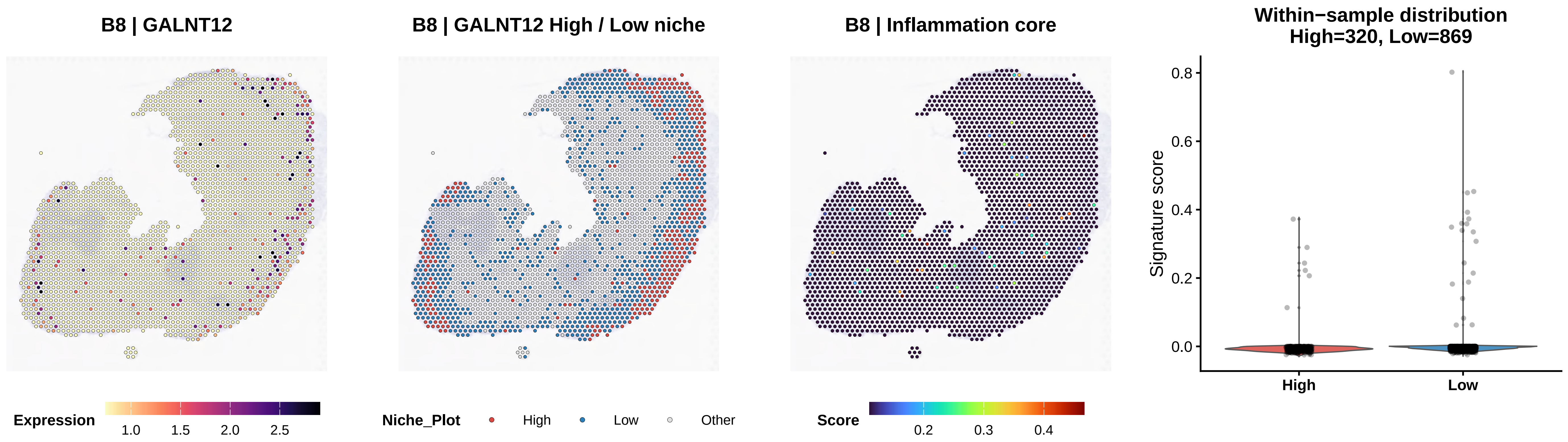

B8 | GALNT12
B8 | GALNT12 High / Low niche
B8 | Inflammation core
Within−sample distribution
High=320, Low=869
Expression
1.0
1.5
2.0
2.5
Niche_Plot
High
Low
Other
Score
0.2
0.3
0.4
Signature score
0.0
0.2
0.4
0.6
0.8
High
Low

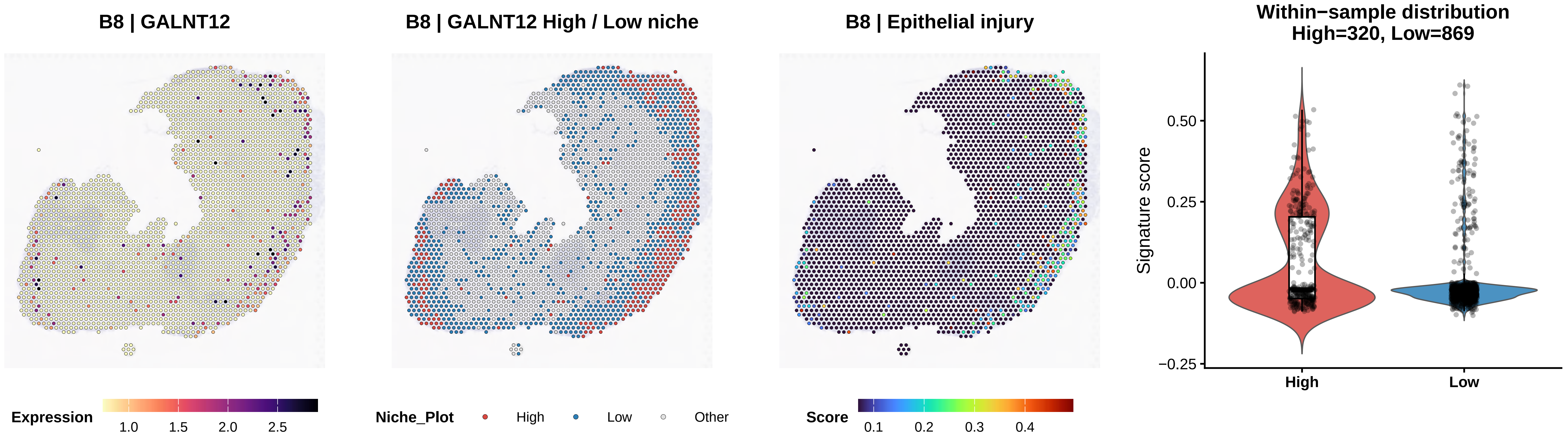

B8 | GALNT12
B8 | GALNT12 High / Low niche
B8 | Epithelial injury
Within−sample distribution
High=320, Low=869
Signature score
0.50
0.25
0.00
−0.25
High
Low
Expression
1.0
1.5
2.0
2.5
Niche_Plot
High
Low
Other
Score
0.1
0.2
0.3
0.4

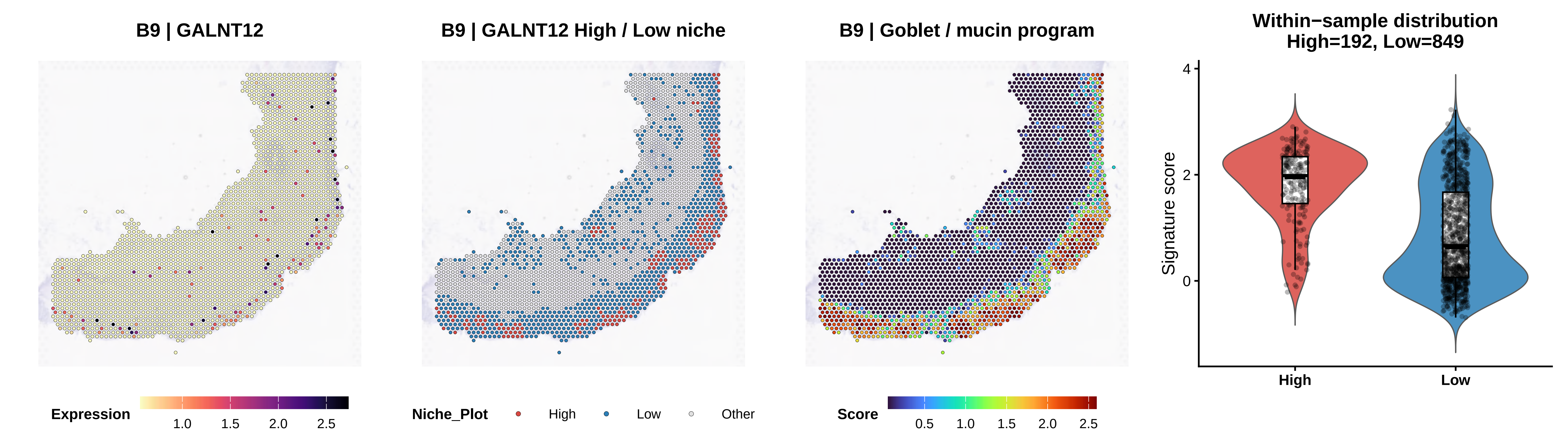
B9 | GALNT12
B9 | GALNT12 High / Low niche
B9 | Goblet / mucin program
Within−sample distribution
High=192, Low=849
Signature score
4
2
0
High
Low
Expression
1.0
1.5
2.0
2.5
Niche_Plot
High
Low
Other
Score
0.5
1.0
1.5
2.0
2.5

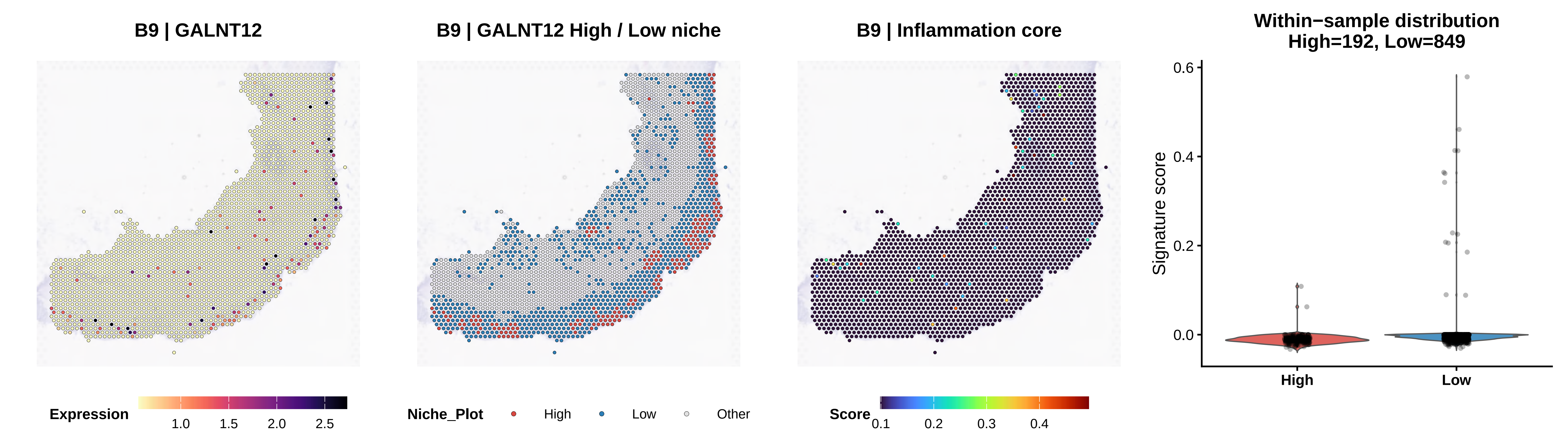
B9 | GALNT12
B9 | GALNT12 High / Low niche
B9 | Inflammation core
Within−sample distribution
High=192, Low=849
Signature score
0.6
0.4
0.2
0.0
High
Low
Expression
1.0
1.5
2.0
2.5
Niche_Plot
High
Low
Other
Score
0.1
0.2
0.3
0.4

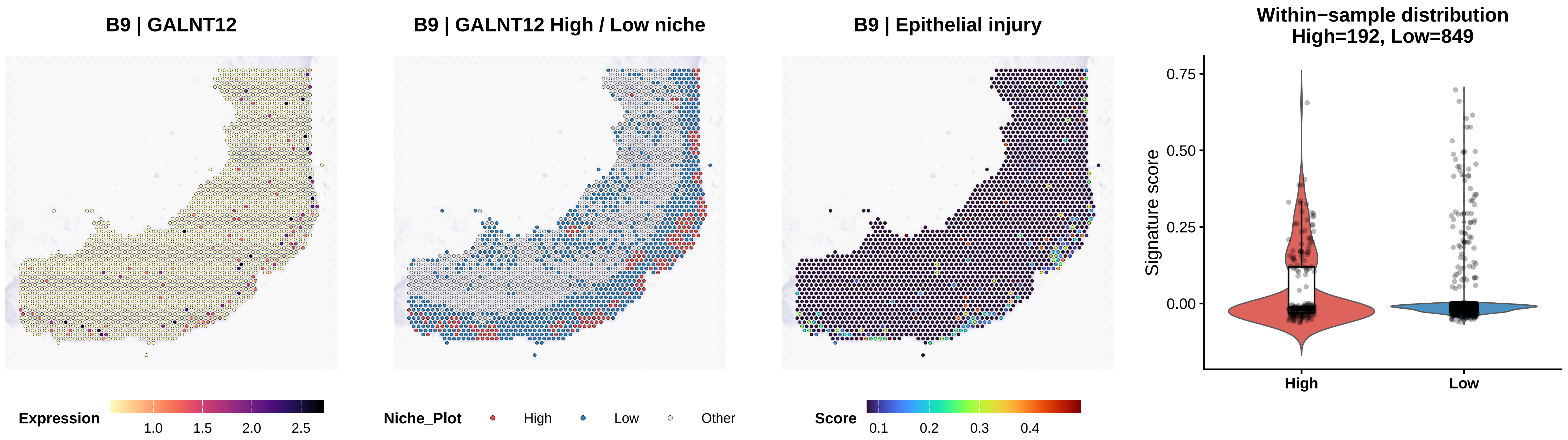
B9 | GALNT12
B9 | GALNT12 High / Low niche
B9 | Epithelial injury
Within−sample distribution
High=192, Low=849
Expression
1.0
1.5
2.0
2.5
Niche_Plot
High
Low
Other
Score
0.1
0.2
0.3
0.4
Signature score
0.75
0.50
0.25
0.00
High
Low

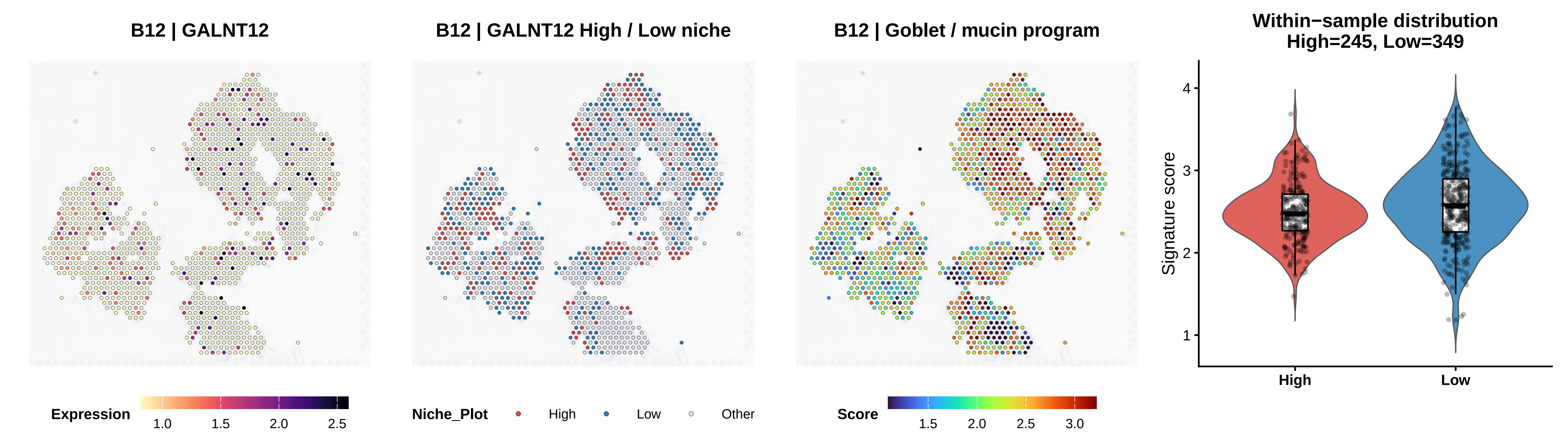
B12 | GALNT12
B12 | GALNT12 High / Low niche
B12 | Goblet / mucin program
Within−sample distribution
High=245, Low=349
Expression
1.0
1.5
2.0
2.5
Niche_Plot
High
Low
Other
Score
1.5
2.0
2.5
3.0
Signature score
1
2
3
4
High
Low

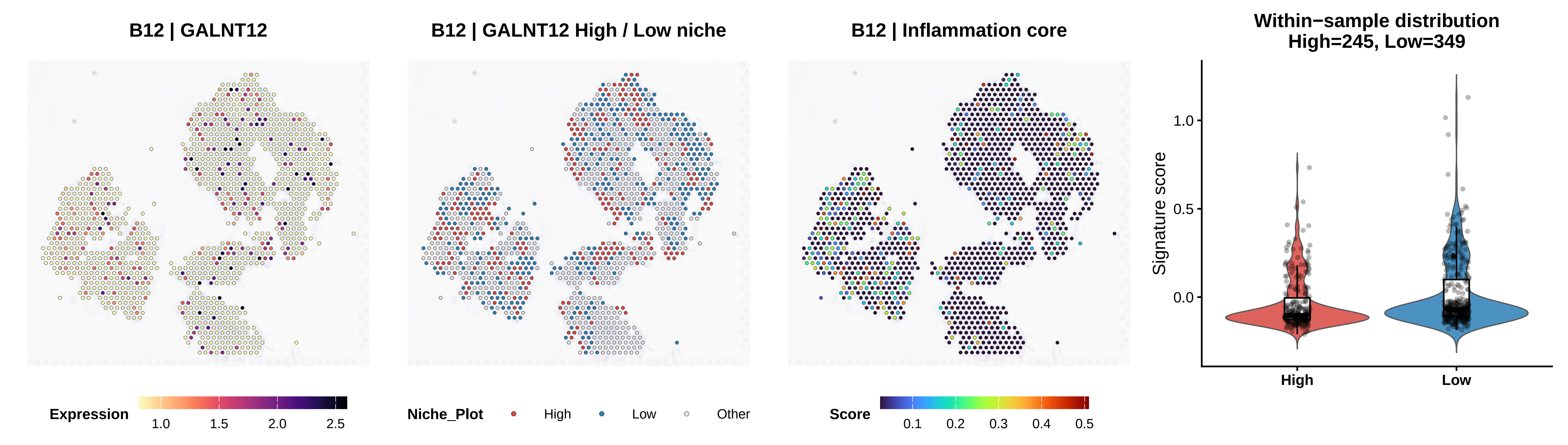
B12 | GALNT12
B12 | GALNT12 High / Low niche
B12 | Inflammation core
Within−sample distribution
High=245, Low=349
Expression
1.0
1.5
2.0
2.5
Niche_Plot
High
Low
Other
Score
0.1
0.2
0.3
0.4
0.5
Signature score
1.0
0.5
0.0
High
Low

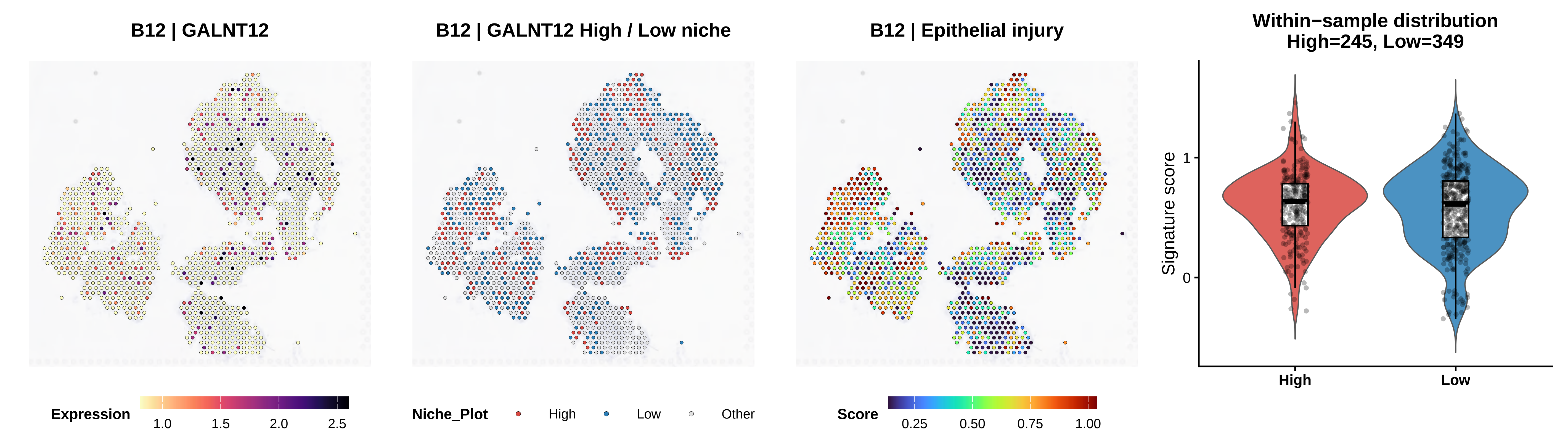
B12 | GALNT12
B12 | GALNT12 High / Low niche
B12 | Epithelial injury
Within−sample distribution
High=245, Low=349
Expression
1.0
1.5
2.0
2.5
Niche_Plot
High
Low
Other
Score
0.25
0.50
0.75
1.00
Signature score
1
0
High
Low

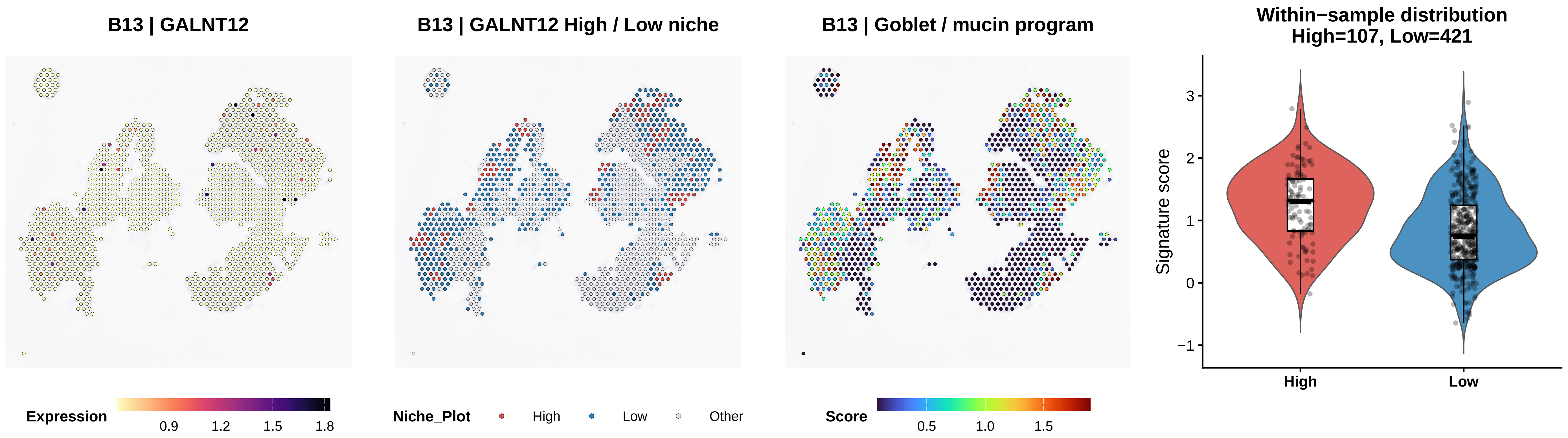

B13 | GALNT12
B13 | GALNT12 High / Low niche
B13 | Goblet / mucin program
Within−sample distribution
High=107, Low=421
Expression
0.9
1.2
1.5
1.8
Niche_Plot
High
Low
Other
Score
0.5
1.0
1.5
Signature score
3
2
1
0
−1
High
Low

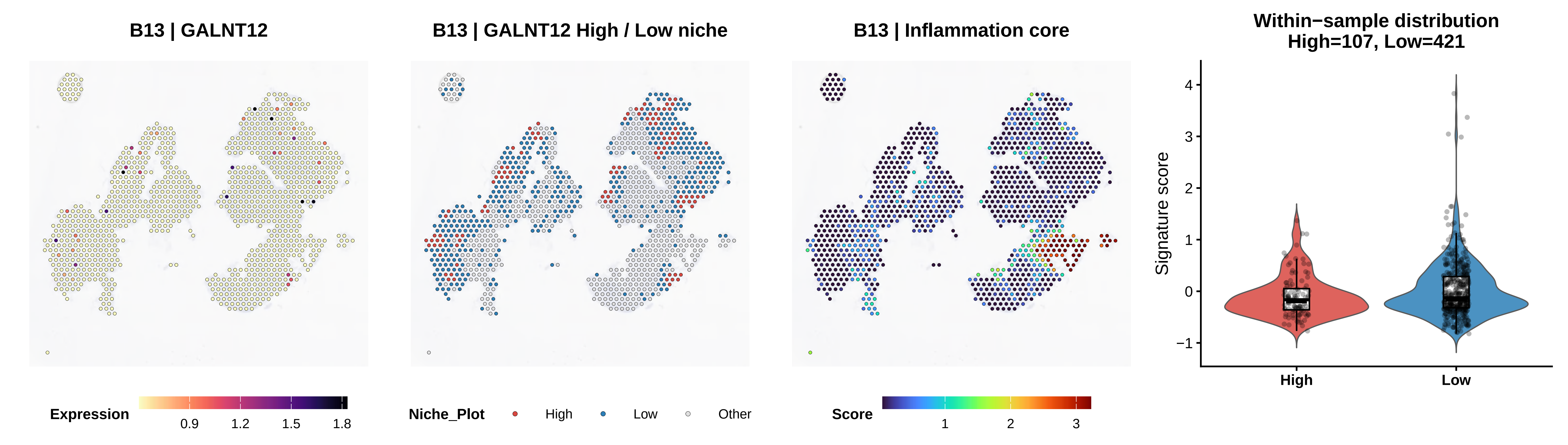
B13 | GALNT12
B13 | GALNT12 High / Low niche
B13 | Inflammation core
Within−sample distribution
High=107, Low=421
Signature score
4
3
2
1
0
−1
High
Low
Expression
0.9
1.2
1.5
1.8
Niche_Plot
High
Low
Other
Score
1
2
3

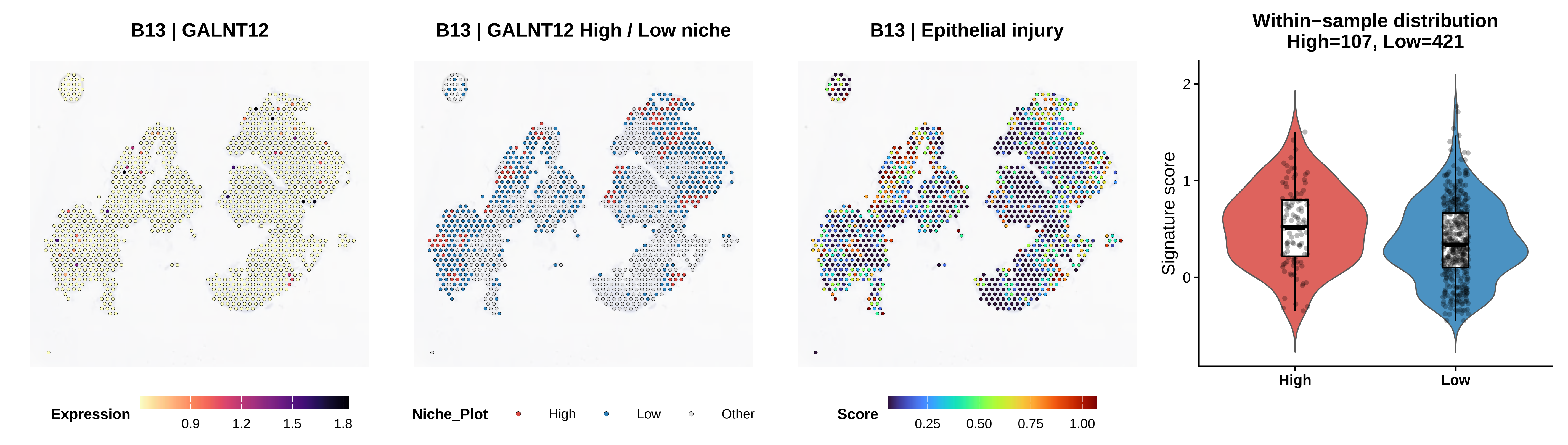
B13 | GALNT12
Expression
0.9
1.2
1.5
1.8
B13 | GALNT12 High / Low niche
Niche_Plot
High
Low
Other
B13 | Epithelial injury
Score
0.25
0.50
0.75
1.00
Within−sample distribution
High=107, Low=421
Signature score
2
1
0
High
Low

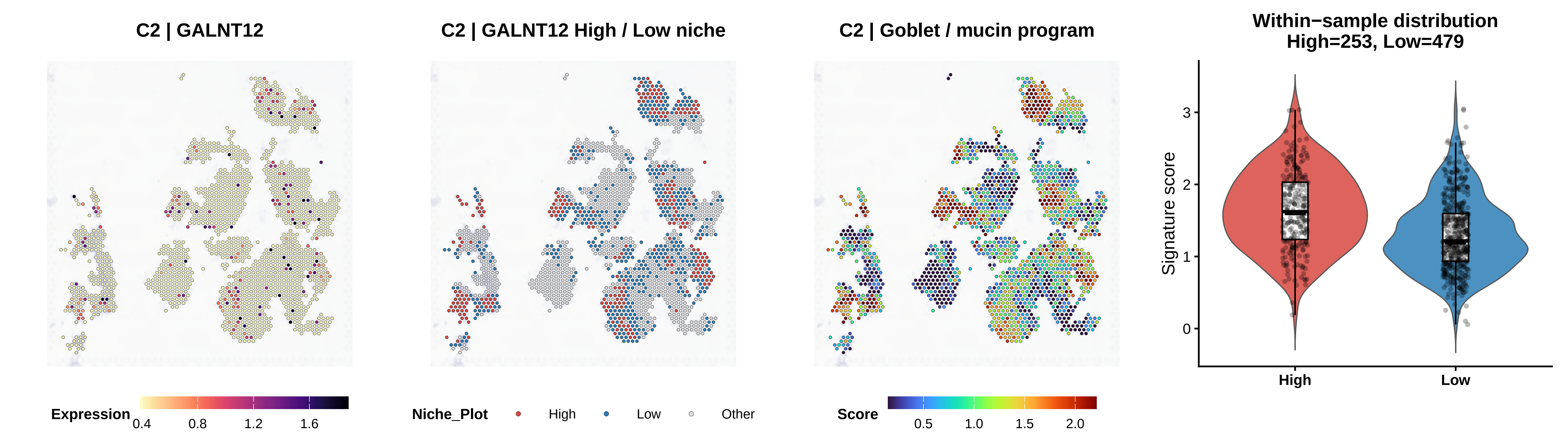
C2 | GALNT12
Expression
0.4
0.8
1.2
1.6
C2 | GALNT12 High / Low niche
Niche_Plot
High
Low
Other
C2 | Goblet / mucin program
Score
0.5
1.0
1.5
2.0
Within−sample distribution
High=253, Low=479
Signature score
0
1
2
3
High
Low

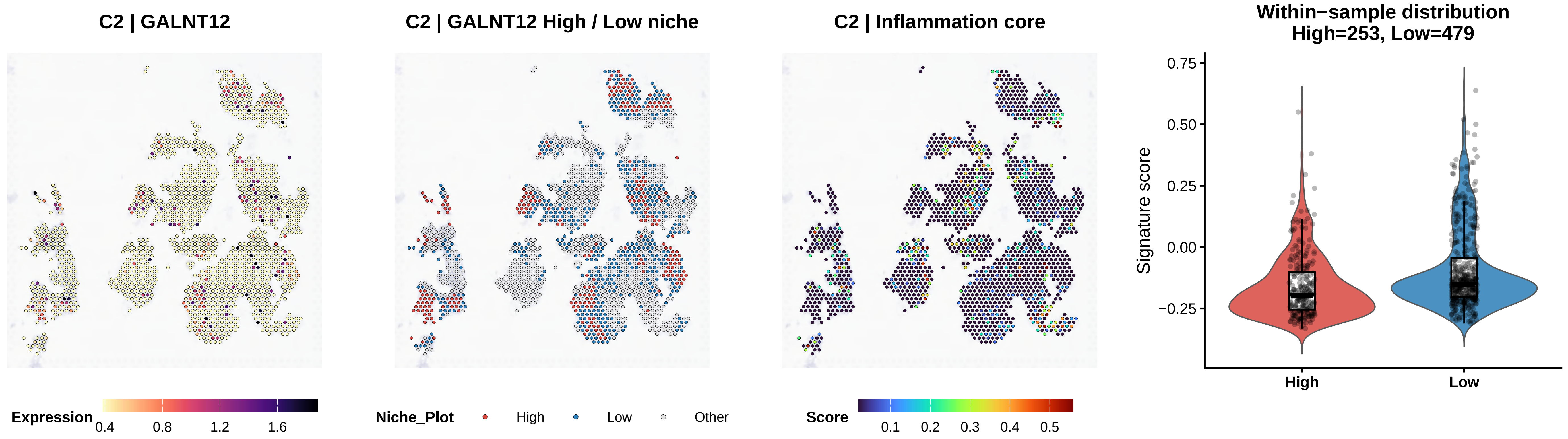
C2 | GALNT12
Expression
0.4
0.8
1.2
1.6
C2 | GALNT12 High / Low niche
Niche_Plot
High
Low
Other
C2 | Inflammation core
Score
0.1
0.2
0.3
0.4
0.5
Within−sample distribution
High=253, Low=479
Signature score
0.75
0.50
0.25
0.00
−0.25
High
Low

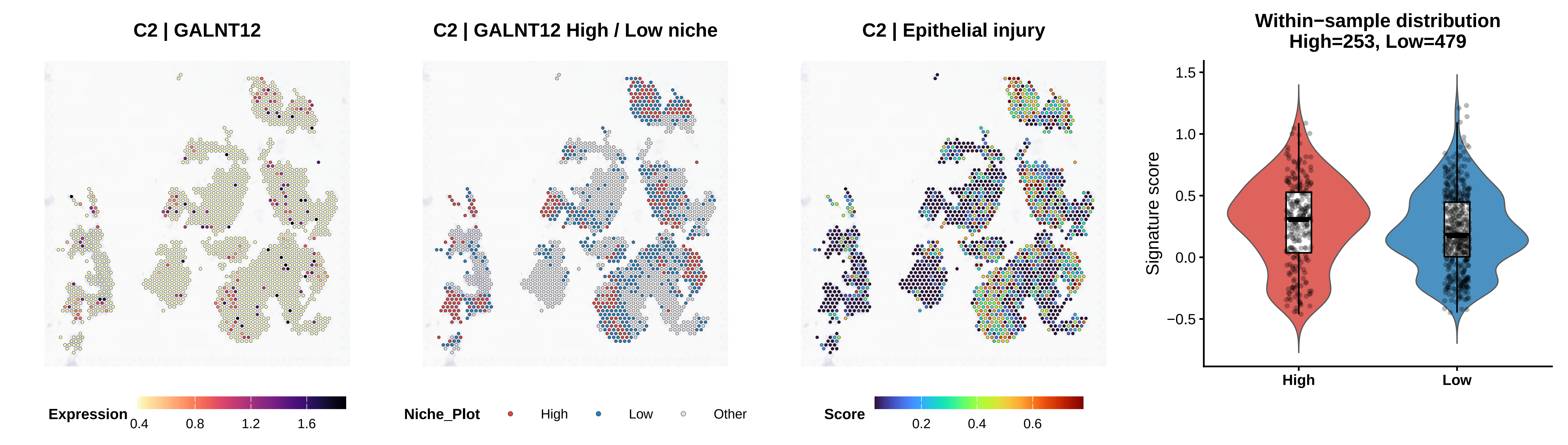
C2 | GALNT12
Expression
0.4
0.8
1.2
1.6
C2 | GALNT12 High / Low niche
Niche_Plot
High
Low
Other
C2 | Epithelial injury
Score
0.2
0.4
0.6
Within−sample distribution
High=253, Low=479
Signature score
1.5
1.0
0.5
0.0
−0.5
High
Low
