# Supplementary figures and images for: Multi-layer transcriptomic analyses identify a mucin-associated epithelial program linked to innate inflammatory injury in ulcerative colitis
Source: Front Immunol. 2026 Jun 3;17:1846672. doi: 10.3389/fimmu.2026.1846672 (PMC13271950; doi:10.3389/fimmu.2026.1846672)

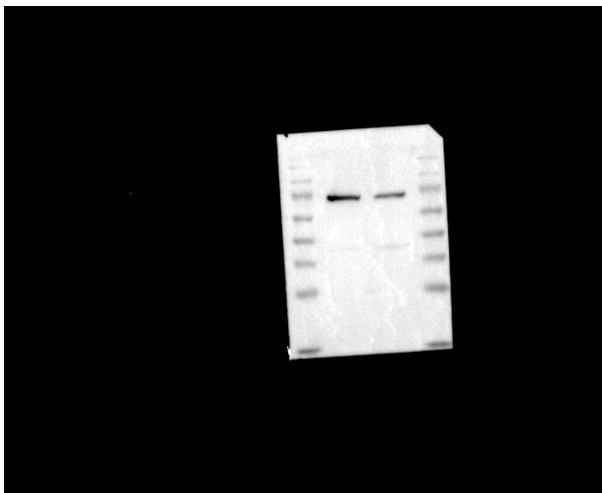

GALNT12

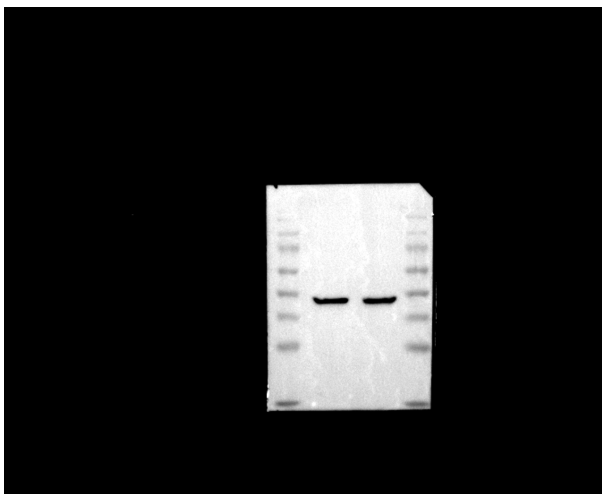

$\beta$ -actin

Supplement: Supplementary Figure 2 — Spatial distribution of GALNT12 and mucin-associated niches across all analyzed colonic biopsy sections. Spatial transcriptomic panels for all seven ulcerative colitis tissue slices (B4, B5, B8, B9, B12, B13, and C2). For each slice, the H&E staining, spatial expression of GALNT12, and the defined mucin-associated epithelial niches are displayed to support the consistency of the spatial findings. [file DataSheet2.pdf]

GSDMD

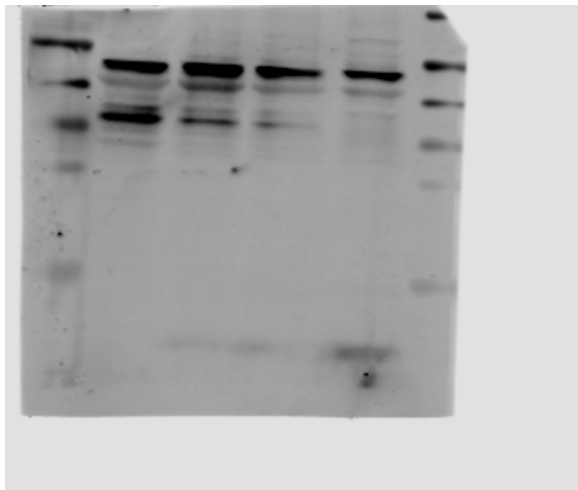

$\beta$ -actin

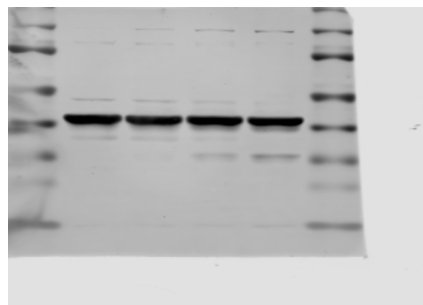

Caspase-1

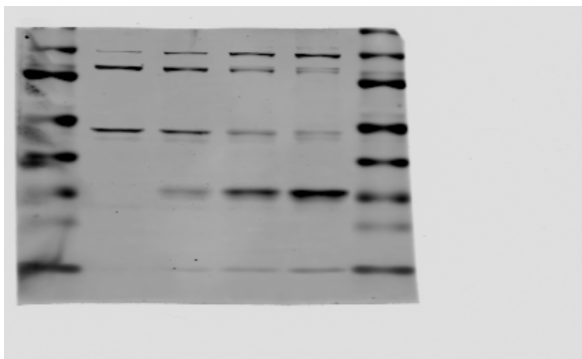

Supplement: Supplementary Figure 3 — Original full-scan images of the western blots. The uncropped blot images corresponding to the experiments shown in Figure 8A are presented. [file DataSheet3.pdf]

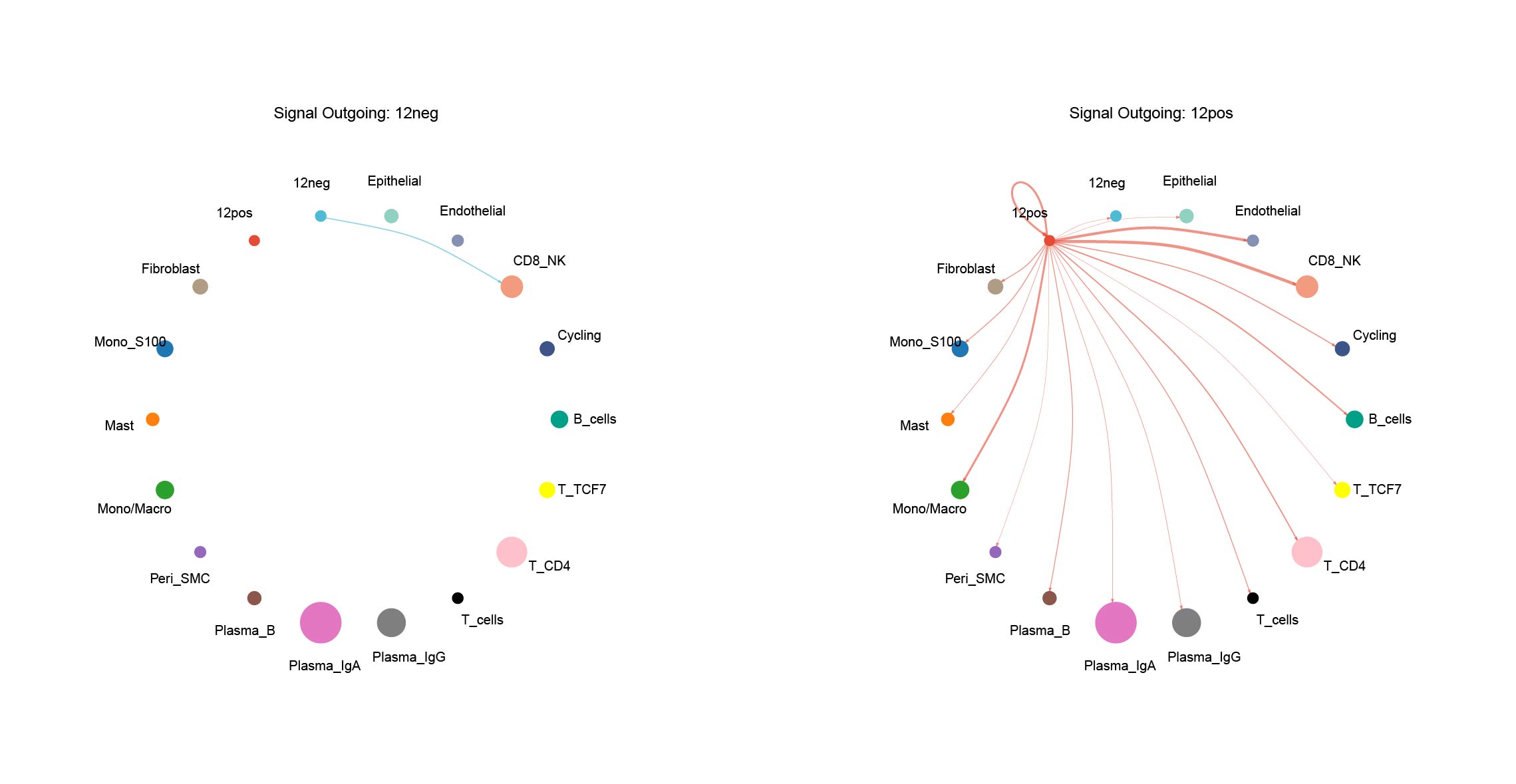

Supplement: Supplementary Figure 4 — Original full-scan images of the western blots. The uncropped blot images corresponding to the experiments shown in Figure 9 are presented. [file Image1.jpeg]
