# Supplementary material for: Multi-layer transcriptomic analyses identify a mucin-associated epithelial program linked to innate inflammatory injury in ulcerative colitis
Source: Front Immunol. 2026 Jun 3;17:1846672. doi: 10.3389/fimmu.2026.1846672 (PMC13271950; doi:10.3389/fimmu.2026.1846672)
Supplement: Supplementary file 5 [file Table1.docx]

**Table S1. List of candidate genes related to mucin-type O-glycosylation.**

| ST3GAL1 |
| --- |
| ST6GALNAC2 |
| ST6GAL1 |
| GALNT16 |
| C1GALT1 |
| GALNTL5 |
| GALNT7 |
| ST3GAL4 |
| GALNT18 |
| GALNT3 |
| MUC5B |
| A4GNT |
| B4GALT6 |
| GALNT12 |
| GCNT7 |
| ST3GAL3 |
| GALNT8 |
| GALNT15 |
| GALNT5 |
| ST6GALNAC4 |
| GALNT6 |
| GCNT3 |
| CHST4 |
| GALNT1 |
| GALNT2 |
| GALNT13 |
| MUC4 |
| B3GNT7 |
| ST3GAL2 |
| GALNT14 |
| B4GALT5 |
| GALNT10 |
| MUC15 |
| MUC17 |
| MUC3A |
| B3GNT2 |
| C1GALT1C1 |
| MUC7 |
| MUCL1 |
| MUC13 |
| GALNTL6 |
| B3GNTL1 |
| B3GNT4 |
| B3GNT5 |
| GCNT4 |
| MUC20 |
| B3GNT8 |
| GALNT11 |
| B3GNT3 |
| MUC16 |
| GALNT9 |
| ST6GALNAC3 |
| MUC6 |
| GALNT17 |
| MUC1 |
| GCNT1 |
| B3GNT6 |
| MUC21 |
| MUC12 |
| MUC5AC |
| B3GNT9 |
| GALNT4 |
| MUC2 |
| ST6GALNAC1 |
| B3GALT5 |
| ERN2 |
| XBP1 |
| AGR2 |
| SPDEF |
| ATOH1 |
| KLF4 |
| TFF3 |
| FCGBP |
| CLCA1 |
| RETNLB |
| IL22RA1 |
| STAT3 |
| FUT2 |
| FUT3 |
| FUT8 |

**Table S2. Detailed information of reagents and resources for in vitro.**

| **Reagent / Antibody / Kit** | **Manufacturer** | **Country** |
| --- | --- | --- |
| McCoy’s 5A medium; Fetal bovine serum (FBS) | Procell | China |
| Penicillin-streptomycin; RIPA lysis buffer | Beyotime | China |
| GALNT12-targeting siRNA; Negative control siRNA | GenePharma | China |
| Recombinant human TNF-α | Novoprotein | China |
| HiScript III RT SuperMix; ChamQ Universal SYBR qPCR Master Mix | Vazyme | China |
| Human IL-8, IL-6, CCL2, IL-1β, and IL-18 ELISA kits | Elabscience | China |
| LDH Cytotoxicity Assay Kit | Beyotime | China |
| Anti-GALNT12 and Anti-β-actin primary antibodies | Proteintech | USA |
| Lipofectamine 3000; TRIzol Reagent | Thermo Fisher | USA |
| Anti-Caspase-1 antibody | Abcam | UK |
| Anti-GSDMD antibody | Abiowell | China |

**Table S3. Oligonucleotide primer sequences used for RT-qPCR analysis**

| **Gene** | **Forward primer (5'-3')** | **Reverse primer (5'-3')** |
| --- | --- | --- |
| *GALNT12* | CCAACAAGAGAGAGGGCCTG | CGGAGTTCCCCAGGTATTCG |
| *IL8* | GAGAGTGATTGAGAGTGGACCAC | CACAACCCTCTGCACCCAGTTT |
| *OCLN* | ATGGCAAAGTGAATGACAAGCGG | CTGTAACGAGGCTGCCTGAAGT |
| *TJP1* | GTCCAGAATCTCGGAAAAGTGCC | CTTTCAGCGCACCATACCAACC |
| *MUC2* | ACTCTCCACACCCAGCATCATC | GTGTCTCCGTATGTGCCGTTGT |
| *ACTB* | CACCATTGGCAATGAGCGGTTC | AGGTCTTTGCGGATGTCCACGT |
